# Discovery of flat-band 2D materials via physics-informed scoring and structure-based learning

**DOI:** 10.1126/sciadv.aea3611

**Published:** 2026-07-08

**Authors:** Xiangwen Wang, Yihao Wei, Anupam Bhattacharya, Qian Yang, Artem Mishchenko

**Affiliations:** Department of Physics and Astronomy, The University of Manchester, Manchester M13 9PL, UK.

## Abstract

Flat electronic bands are a fertile ground for exotic quantum phenomena, from unconventional superconductivity to fractional topology. However, their rarity and the reliance on density functional theory (DFT) calculations for identification have limited systematic exploration across large material spaces. Here, we report the discovery of multiple previously unrecognized flat-band two-dimensional materials through a structure-informed, data-driven framework. We introduce a physically motivated flatness score that combines band dispersion and density-of-states features, enabling algorithmic labeling of known materials without requiring manual inspection. This score is then used to train a multimodal deep learning model that predicts flat-band propensity directly from atomic structure. Applied to more than 10,000 unlabeled materials, our framework identifies numerous flat-band candidates. Among the top-scoring structures (flatness score higher than 0.9), DFT calculations on materials with kagome-like lattices confirm a flat-band prediction accuracy of 98%, demonstrating the ability to discover topologically nontrivial systems. By bypassing the need for precomputed band structures, our approach enables large-scale, interpretable screening and expands the design space for correlated quantum phases in low-dimensional systems.

## Teaser

A data-driven framework uncovers flat-band 2D materials with 98% prediction accuracy, verified by quantum calculations.

## INTRODUCTION

Two-dimensional (2D) materials that host flat electronic bands have become a prime platform for investigating strongly correlated quantum states. When the bandwidth collapses, the electronic kinetic energy is quenched and electron-electron interactions dominate, enabling phenomena such as unconventional superconductivity, ferromagnetism, topological order, and fractional quantum Hall states (*1*–*9*). Representative systems include kagome lattice compounds, Lieb lattice structures, magic-angle twisted bilayer graphene, other moiré superlattices, and other non-moiré materials (*10*–*21*). These examples underscore a central principle: band flatness is a crucial driver of correlated phases.

Flat bands, however, arise from diverse mechanisms. Some are trivial, originating from localized atomic orbitals, whereas others result from interference among extended states and may carry nontrivial topology. Distinguishing these cases generally requires detailed analysis of the electronic structure, which limits the scalability of existing discovery workflows. (*22*) As a result, there is a growing need for general and efficient methods that can detect flat-band features across large databases, regardless of their microscopic origin.

Despite their significance, discovering topological flat-band materials remains difficult because density functional theory (DFT) calculations are computationally intensive. A large variety of previous approaches are based on DFT-calculated bandwidth. For example, Liu *et al.* (*23*) screened band structure data from 2DMatPedia (*24*); Regnault *et al.* (*25*) and Duan *et al.* (*26*) applied bandwidth and density of state (DOS)–based screening on materials from the Inorganic Crystal Structure Database (ICSD) (*27*, *28*) after high-throughput DFT calculations. These filters all hinge on manually defined selection criteria, such as an arbitrary bandwidth cutoff, which introduces bias. Bhattacharya *et al.* (*29*) replaced explicit thresholds with a convolutional neural network that classifies DFT-computed electronic band structure images, but their approach still depends on costly DFT simulations and manually labeled data. However, as all these methods rely on prior DFT calculations, scaling them to unexplored chemical spaces becomes prohibitively expensive. Approaches that forgo DFT are rarer: Neves *et al.* (*30*) identified low-dispersion motifs by constructing tight-binding models across the Materials Project database, but the method rests on strong assumptions in the tight-binding parametrization and requires well-defined crystal nets, limiting its generality.

Although data-driven models offer a powerful alternative to traditional electronic structure calculations, most machine learning studies still predict only scalar electronic properties, typically the bandgap or averaged DOS, from crystal structure (*31*, *32*). As a result, models built on handcrafted descriptors (*33*), convolutional neural networks (*34*), graph neural networks, transformer architectures (*35*), and language models based on textual representations (*36*, *37*) have achieved impressive numerical accuracy. However, scalar quantities like bandgap capture only coarse features of the band structure and miss the nuances critical for identifying flat bands or correlated topological phases. Despite the importance of flat bands, there is currently no general-purpose framework that can discover such phases directly from atomic structures.

In this work, we introduce a scalable and interpretable framework for discovering flat bands in 2D materials via crystal structure–informed learning, without requiring any precomputed electronic structure. By integrating high-throughput inference, sublattice-informed filtering, and embedding-space analysis, our method identifies previously unrecognized flat-band candidates from large-scale materials databases such as Nb_3_TeI_7_, Cu_3_AsO_4_, and Cu_3_SbO_4_ that exhibit fragile topological features near the Fermi level, as confirmed by DFT and band representation analysis. Central to our approach is a physics-motivated flatness score, derived from electronic band dispersion and DOS features, which serves as an interpretable supervision signal grounded in materials physics. This score is predicted directly from atomic structures using a multimodal deep learning model trained on aligned graph and text encoders. Scalability is achieved by applying the model to more than 10,000 candidate structures without requiring explicit band structure inputs, enabling efficient screening across large chemical spaces. Our framework enables direct discovery of flat-band candidates from atomic structure alone, eliminating the need for electronic structure inputs and allowing access to unexplored materials spaces. Using this approach, we identified multiple previously unreported flat-band 2D materials with DFT-confirmed fragile topology, demonstrating its power as a discovery tool for correlated quantum phases.

## RESULTS

**Fig. 1.**
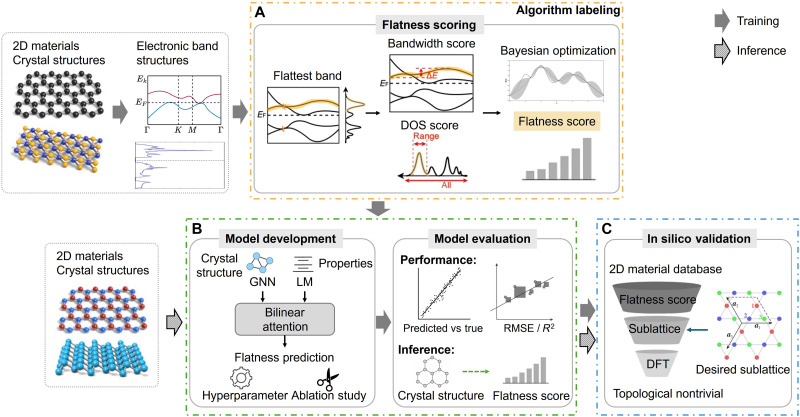
Framework for automatic labeling and identification of flat-band phases in 2D materials using structure-informed algorithms. (**A**) Dataset labeling and flatness scoring. A composite flatness score combining bandwidth and DOS characteristics is used to automatically label training data based on electronic band structures. (**B**) Multimodal model development. A multimodal model trained on structural and textual features predicts flatness scores from crystal structure alone, with performance validated on labeled data. (**C**) In silico validation. The high-scoring candidates are filtered by kagome-like sublattice motifs and validated via DFT calculations.

Figure 1 outlines the workflow for large-scale discovery of flat-band 2D materials. First, materials with known electronic structures are labeled via a flatness score combining band dispersion and DOS characteristics, capturing essential spectral signatures of flat-band behavior (Fig. 1A). This algorithmic labeling provides a continuous and physically grounded supervision signal for training a multimodal deep learning model on atomic structural inputs, combining geometric and contextual representations (Fig. 1B). The trained model is then applied to a broad set of unlabeled 2D materials to rank candidates by predicted flatness. High-scoring structures are further screened through sublattice motif analysis and validated by DFT calculations, confirming the presence of topologically nontrivial flat bands in multiple cases (Fig. 1C). By learning structure-property relationships grounded in physically defined flatness criteria, the framework enables large-scale screening of candidate materials without electronic structure inputs, while preserving meaningful connections to underlying band topology.

### High-throughput scoring of band flatness in 2D materials

To systematically identify flat-band features in 2D materials, we constructed a dual-metric framework to automatically generate flatness scores based on electronic band dispersion and DOS characteristics, without manual annotation. We applied this procedure to the 2DMatPedia database (*24*), which provides computed electronic structures for around 5100 2D materials.

For each material, we first detect band crossings, then divide the band structure—between consecutive high-symmetry points—into continuous, noncrossing segments. These segments are then reconnected at crossing points to form multiple end-to-end combinations, from which the one with the narrowest energy span is chosen as the representative band of the material.

We define a momentum-space flatness score, $S_{\text{bandwidth}}$, to quantify the dispersion of the material. Specifically, we first compute the energy span of the previously identified representative band, and then map the raw value to the interval [0,1] using a cosine transformation that penalizes broader bands: A perfectly flat segment approaches one, while highly dispersive bands approach zero. Building on this definition, we introduce a tunable threshold $\omega_{\text{max}}$ to specify the maximum energy span that is still considered “flat”; the energy span wider than this limit automatically receive zero scores. We intentionally avoid an overly strict cutoff (for example, 0.05 eV), which would shrink the training set and reduce the problem to a binary classification. By retaining a continuous, regression-style score, the model preserves subtle gradations of flatness and enables fine-grained prioritization of candidate materials.

To complement this dispersion-based metric, we define a DOS measure, $S_{\text{DOS}}$, which recognizes the pronounced DOS peaks typical of flat-band systems (*38*, *39*). For representative band of every material, we center a window of width $\omega_{\text{max}}$ on the band midpoint, calculate the mean DOS inside that window, and compare it with the DOS of the fixed reference range of [−5 eV, 5 eV] (*25*). The contrast is then normalized to [0,1], where higher values indicate sharper peaks. A saturation function mitigates the influence of low background DOS, stabilizing the score.

The overall flatness metric, $S_{\text{total}}$, combines $S_{\text{bandwidth}}$ and $S_{\text{DOS}}$ under a “both-must-be-high” principle: if $S_{\text{bandwidth}}$ falls below $\omega_{\text{max}}$, $S_{\text{total}}$ is set to zero regardless of the DOS peak. Rather than manually defining the combination weights, we learn them via Bayesian Optimization (BO), letting the data tune the parameters to best reflect the underlying physics.

**Fig. 2.**
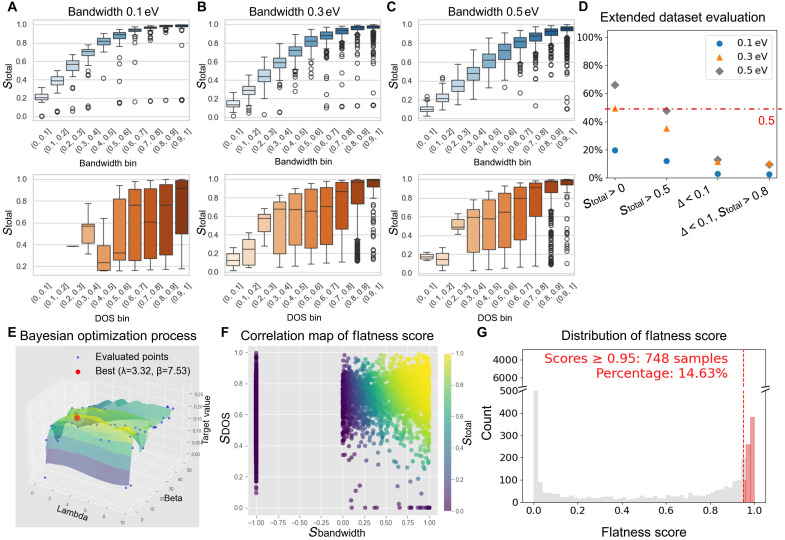
Flatness scoring optimization and analysis of electronic structure data. (**A** to **C**) Box plots of different bandwidth windows (0.1, 0.3, and 0.5 eV) showing the distribution of $S_{\text{total}}$ for samples with $S_{\text{total}}>0$, grouped by intervals of $S_{\text{bandwidth}}$ (top panel) and $S_{\text{DOS}}$ (bottom panel); color intensity reflects the number of samples in each bin to indicate data balance across intervals. (**D**) Evaluation of dataset-level quality metrics across three different bandwidth windows (0.1, 0.3, and 0.5 eV). Δ refers to the absolute difference between the two subscores, defined as $|S_{\text{bandwidth}}-S_{\text{DOS}}|$. (**E**) Bayesian optimization process with evaluated points (blue) and best parameters (red). (**F**) Correlation map of flatness score, $S_{\text{bandwidth}}$, and $S_{\text{DOS}}$, colored by $S_{\text{total}}$. (**G**) Distribution of $S_{\text{total}}$ scores across the whole dataset, with a threshold of 0.95 used to show the top high-quality candidates.

Although our flatness metrics are continuous, their effectiveness depends on the bandwidth threshold $\omega_{\text{max}}$. We evaluated three values, 0.1, 0.3, and 0.5 eV, to examine how different thresholds influence both the score distribution and sample balance. As shown in Fig. 2 (A to C), when $\omega_{\text{max}}$ is set to 0.3 or 0.5 eV, $S_{\text{total}}$ increases more smoothly across binned $S_{\text{bandwidth}}$ and $S_{\text{DOS}}$ values, with more balanced sample distributions as indicated by the color intensity. In contrast, the 0.1 eV setting causes the scores to saturate at low values, resulting in sparsely populated high-score regions and limited usable data coverage. Figure 2D summarizes the proportion of structures meeting increasingly strict quality criteria across the full dataset. These include basic validity ($S_{\text{total}}>0$), high flatness confidence ($S_{\text{total}}>0.5$), structural balance (measured by $\Delta =|S_{\text{bandwidth}}-S_{\text{DOS}}|<0.1$), and a combined constraint that selects only those structures with both high scores and balanced contributions (Δ<0.1 and $S_{\text{total}}>0.8$). While 0.5 eV yields the highest number of candidates, 0.3 eV provides comparable coverage and imposes a stricter physical constraint on band dispersion, enhancing selectivity. In contrast, the 0.1-eV threshold is too stringent, resulting in limited usable data, eliminating most samples under any realistic constraint.

Figure 2E shows the optimized parameter space obtained using a bandwidth threshold of 0.3 eV, where $S_{\text{total}}$ peaks in regions with simultaneously high $S_{\text{bandwidth}}$ and $S_{\text{DOS}}$, while penalizing imbalance. This results in a nontrivial scoring surface that preserves resolution across the dataset and highlights high-quality flat-band candidates. Figure 2F visualizes the joint distribution of $\left(S_{\text{bandwidth}},S_{\text{DOS}}\right)$, color coded by $S_{\text{total}}$. High-scoring samples cluster in the top right, confirming that the learned combination captures the desired trade-off. Entries that exceed the bandwidth threshold ($S_{\text{bandwidth}}=-1$) appear as a vertical strip with $S_{\text{total}}=0$. The overall score distribution in Fig. 2G shows that, while a substantial portion of materials are filtered out, the remaining entries span a broad range. Notably, 748 materials (14.63%) achieve scores above 0.95 and are shortlisted for further theoretical and structural investigation. Results for other thresholds (0.1 and 0.5 eV) are provided in the Supplementary Text (section A).

### Deep learning model for flatness score prediction

#### Multimodal deep learning framework

To directly predict the flatness score from raw material inputs, we constructed a multimodal deep learning framework that integrates structural and textual information of 2D material properties.

**Fig. 3.**
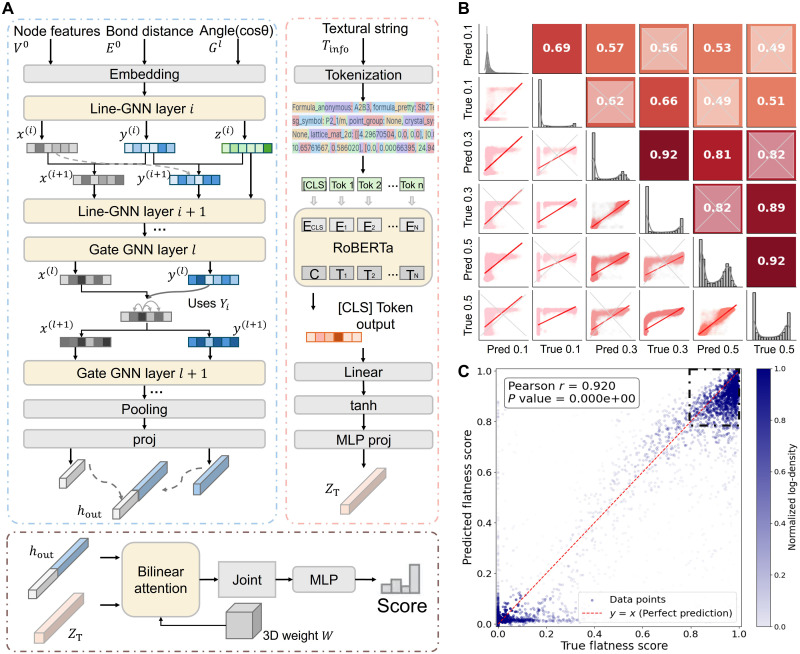
Multimodal architecture for predicting flatness scores and assessing model performance. (**A**) Model overview from crystal structure to flatness score prediction. Atomic structures are encoded using a gated line graph neural network, while textual material descriptions are processed via a RoBERTa encoder. The two modalities are fused through bilinear attention to produce a continuous flatness score. (**B**) Pairwise relationships between predicted and true values across experiments with bandwidth thresholds $\omega_{\text{max}}$ of 0.1, 0.3, and 0.5 eV. Diagonal plots display the distribution of each variable (histogram + KDE). Lower triangle plots show scatter plots with regression lines (red); point transparency reflects density. Upper triangle plots present Pearson correlation coefficients, with background color intensity indicating correlation strength (darker for stronger). Light gray “X” markers denote noncomparable pairs. (**C**) The performance of model trained with bandwidth thresholds $\omega_{\text{max}}$ of 0.3 eV: predicted versus true flatness scores.

The structural encoder is based on the ALIGNN framework (*40*), which augments graph neural networks with line graph–based geometric priors, capturing bond lengths and angular relations through edge-gated message passing. As illustrated in Fig. 3A (left), the model processes atomistic features across stacked Line-GNN and Gate-GNN layers to extract hierarchical descriptors of 2D crystal geometry. In parallel, chemical formulas and structured descriptors are embedded using a transformer-based encoder (Fig. 3A, right), where a pretrained RoBERTa model (*41*) generates contextualized token-level embeddings. These are projected into a fixed-dimensional latent vector. The two modalities are fused via a bilinear attention module, which learns cross-modal interactions between structural and textual embeddings. The final joint representation is used to predict the flatness score. This end-to-end model allows prediction from unprocessed material inputs, eliminating manual feature engineering while capturing both local structural features and semantic material context relevant to flat-band behavior.

#### Model performance on the labeled dataset

After training on the labeled dataset, we systematically evaluated the predictive performance of our multimodal model. Figure 3B presents pairwise comparisons between predicted and true flatness scores across the three bandwidth thresholds (0.1, 0.3, and 0.5 eV), showing both within-threshold accuracy and cross-threshold consistency. Under each individual bandwidth setting, the Pearson correlation coefficients between predicted and true values were 0.66 for 0.1 eV, and 0.92 for both 0.3 and 0.5 eV, indicating comparable predictive accuracy between the latter two, and a substantial improvement over the stricter 0.1-eV threshold. Cross-setting comparisons also show strong correlations, particularly between the 0.3- and 0.5-eV conditions, where the true-value correlation reaches 0.89 and the predicted-value correlation is 0.81. These results suggest that the model outputs are robust to bandwidth variation, with especially high consistency between the 0.3- and 0.5-eV settings. Together, these findings indicate that a threshold of 0.3 eV achieves the same predictive fidelity as 0.5 eV, while avoiding the excessive permissiveness associated with broader thresholds. It thus offers a more desirable trade-off between precision and generalizability. All subsequent analyses are therefore based on models trained under the $\omega_{\text{max}}=0.3$ eV bandwidth setting.

Figure 3C further examines the predictive accuracy under the 0.3-eV setting. The predicted scores exhibit strong agreement with ground truth, with a Pearson correlation of 0.92 and a *P* value effectively equal to zero. Predictions closely follow the identity line, indicating that the model effectively learns the distribution of flatness scores and captures subtle variations across diverse 2D materials. Notably, the model consistently identifies highly flat-band systems, as reflected by the clustering of high-score predictions in the upper right region. To assess the model’s effectiveness in practical screening, we examined the proportion of true flat-band materials (with true scores higher than 0.9) among the top-k candidates. This highlights the model’s ability to reliably prioritize high-quality candidates for flat-band materials discovery. In addition to correlation analysis, we report absolute error metrics to quantify predictive accuracy. The model achieves a training root mean square error (RMSE) of 0.099 (*R*^2^ = 0.939) and a validation RMSE of 0.281 (*R*^2^ = 0.534) under the $\omega_{\text{max}}=0.3$ eV setting. These error levels provide sufficient resolution for reliable ranking and prioritization of candidate materials. The reliability of the predicted score values themselves is assessed through a calibration analysis, as detailed in the Supplementary Text (section B). 

#### Visualization of structural embeddings

**Fig. 4.**
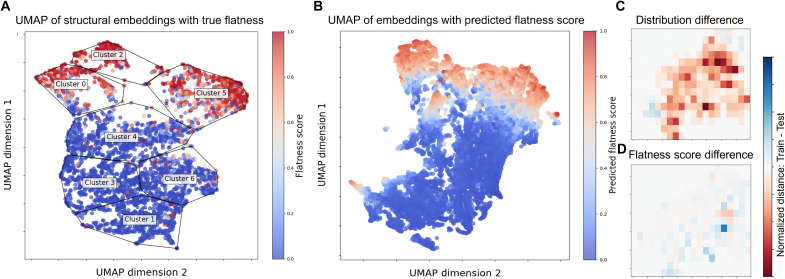
Clustering visualization of structural embedding space and dataset generalization. (**A**) UMAP projection of structural embeddings from the training dataset (2DMatPedia), colored by true flatness score. (**B**) UMAP projection of structural embeddings from the inference dataset (C2DB), colored by predicted flatness score. (**C**) Distribution difference between training and inference datasets across the embedding space. Red regions indicate areas where C2DB materials are overrepresented, pointing to structural motifs absent during training. (**D**) Flatness score difference between training and inference datasets, aggregated over the same embedding regions.

To interpret the learned structure-property relationship beyond scalar predictions, we analyzed the structural embeddings produced by the graph encoder. Using uniform manifold approximation and projection (UMAP) (*42*), we visualized the latent space learned from the training set (2DMatPedia) in Fig. 4A. Materials with similar flatness scores form distinct clusters, with low- and high-flatness systems occupying well-separated regions. This indicates that the model organizes atomic structures according to latent features relevant to flat-band formation, providing an interpretable geometry-aware fingerprint. We further performed a global leave-one-cluster-out evaluation to assess out-of-distribution generalization; full results are reported in the Supplementary Text (section B).

We then applied the model to an inference set of 13,724 materials from the Computational 2D Materials Database (C2DB) (*43*), where flatness scores were predicted directly from atomic structure without using any band structure information. As shown in Fig. 4B, the inferred embeddings also show clear clustering by predicted flatness, confirming that the model generalizes effectively to previously unseen structures.

To assess how the embedding space shifts between training and inference, we discretized the UMAP projections into spatial grid cells and compared the relative sample density (Fig. 4C) and the average flatness score difference (Fig. 4D) between the two datasets. Notably, several peripheral and central regions are dominated by C2DB structures, reflecting underrepresented motifs in the training data. However, the flatness score remains stable across most of the embedding space, with only localized deviations. This suggests that the model retains predictive robustness even when extrapolating to out-of-distribution geometries. A complete catalog of high-scoring C2DB materials, including predicted score, cluster assignment, and kagome flag, is provided on our GitHub repository.

### Flat-band candidate discovery and DFT validation

#### Screening on labeled and inference datasets

**Fig. 5.**
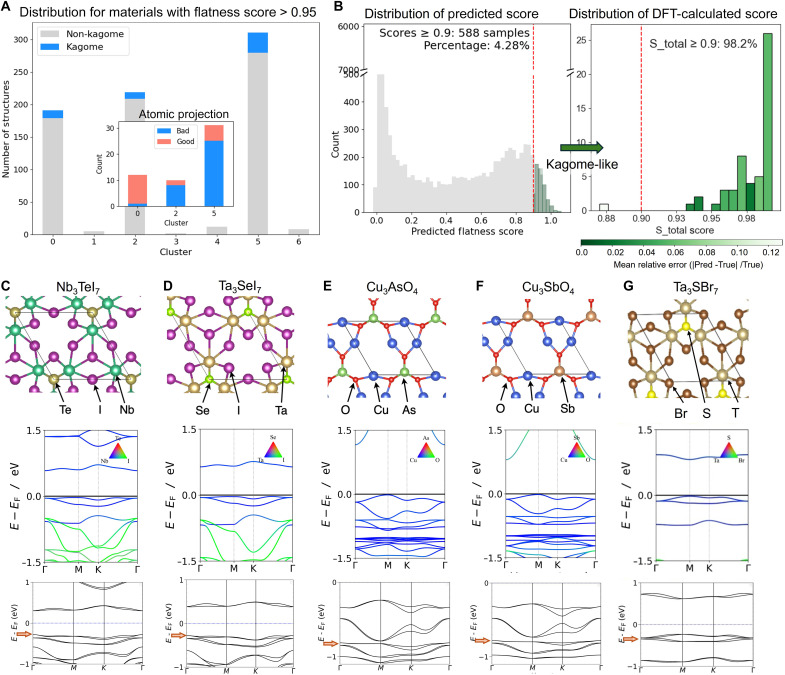
Screening and first-principles validation of materials with high flatness score. (**A**) Cluster-wise distribution of screened flat-band materials from the labeled 2dmatpedia database. Inset: Atomic projection quality for selected clusters (0, 2, and 5). (**B**) Screening pipeline and DFT validation for high-scoring candidates from the unlabeled C2DB database. Left: Predicted flatness score distribution for the full dataset, with structures scoring ≥0.9 selected. Right: DFT-derived flatness scores ($S_{\text{total}}$) for kagome-like candidates, 98.2% of which remain ≥0.9. (**C** to **G**) Crystal structure and DFT-calculated band structures of selected flat-band candidates, showing the unit cell (top), projected bands without SOC (middle), and full bands with SOC (bottom). Flat bands (red arrows) near the Fermi level are predominantly supported by transition metal atoms forming the kagome motif and remain isolated under SOC, exhibiting fragile topological features. Nb_3_TeI_7_: flat band primarily from Nb orbitals, bandwidth of the flattest band: 0.09 eV, distance to $E_{F}$: 0.32 eV. Ta_3_SeI_7_: similar kagome-derived flat band formed by Ta atoms, bandwidth: 0.10 eV, distance to $E_{F}$: 0.31 eV. Cu_3_AsO_4_: Cu-based kagome structure with flat band persisting under SOC, bandwidth: 0.05 eV, distance to $E_{F}$: 0.83 eV. Cu_3_SbO_4_: chemically similar to Cu_3_AsO_4_, exhibiting analogous flat-band characteristics, bandwidth: 0.03 eV, distance to $E_{F}$: 0.80 eV. Ta_3_SBr_7_: SOC-isolated flat bands mainly supported by Ta orbitals, bandwidth: 0.07 eV, distance to $E_{F}$: 0.38 eV.

To examine whether our flatness score reflects meaningful structural signatures, we first analyzed materials in the labeled 2DMatPedia dataset using the calculated flatness scores. We choose materials with scores above 0.95 and map their locations in the UMAP projection (Fig. 5A). These high-flatness materials formed distinct clusters, within which we focused on structures containing kagome motifs due to their known propensity for hosting topological flat bands. We then examined the orbital-projected band structures of these candidates and retained only those where the near-Fermi flat band is predominantly derived from atoms in the kagome sublattice (see the inset of Fig. 5A). Materials failing this atomic projection criterion were discarded. A complete catalog of high flatness score materials, including cluster assignment, kagome-sublattice annotation, and orbital projections, is available on our Github repository. To simplify topological characterization, we excluded materials labeled as “magnetic” in the 2DMatPedia database, avoiding the additional complexity introduced by magnetic ordering. The screening distilled our pool to 15 high-confidence flat-band candidates, primarily located in clusters 0, 2, and 5.

To assess the model’s transferability, we applied it to the structurally diverse but electronically unlabeled C2DB database (*43*). Flatness scores were predicted for all materials using only their atomic structures as input. We then selected candidates with predicted scores above 0.90 and applied motif-based filtering to retain those containing kagome-like structural units, yielding 55 nonmagnetic materials. For these selected candidates, we performed DFT calculations to obtain their electronic band structures. The flatness score was recalculated using the same physics-informed metric defined during model training. As shown in Fig. 5B, 98% of these candidates retained high DFT-derived flatness scores above 0.90, confirming that the model reliably identifies flat band–prone structures even in previously unseen chemical spaces. This result demonstrates both the predictive robustness and practical utility of our structure-informed discovery pipeline.

#### DFT validation and fragile topology

The top-ranked materials were investigated by DFT calculations to verify the presence of topological flat bands. One representative case is Nb_3_TeI_7_ (2dmatpedia ID 2dm-3841), shown in Fig. 5C. Its breathing kagome Nb sublattice hosts almost dispersionless band (ω≈0.09 eV) near the Fermi level. Inclusion of spin-orbit coupling (SOC) separates the flat band from its neighbor and lifts the spin degeneracy everywhere except at Γ point. Topological quantum chemistry analysis was performed using elementary band representations (EBRs) (*44*, *45*). Nb_3_TeI_7_ belongs to layer group 69 (P3m1). The spin-orbit-split doublet transforms as $\Gamma_{4}⊕\Gamma_{5}$, $K_{4}⊕K_{5}$, and $M_{3}⊕M_{4}$ at the high symmetry points (HSPs), which corresponds to EBR combination $\left[{1b|}^{1}{\bar{E}}^{2}\bar{E}↑G\left(2\right)\right]⊕\left[1b|{\bar{E}}_{1}↑G(2)\right]⊖\left[1a|{\bar{E}}_{1}↑G\left(2\right)\right]$. Because the little-group irreducible representations at HSPs cannot be expressed as a positive sum of the layer-group EBRs but as a difference between them, fragile topology is confirmed, akin to the flat bands in magic-angle twisted bilayer graphene (*1*, *3*, *46*). The topological flat bands are supported by Nb atoms forming a breathing kagome sublattice structure, as a result of destructive interference of wave functions associated with this geometry. Notably, a chemically similar system, Nb_3_Cl_8_, has been experimentally demonstrated to have topological flat bands (*47*).

Several additional high-flatness candidates from 2DMatPedia display the same fragile kagome-derived topology. Ta_3_SeI_7_ (2dm-5470) (Fig. 5D), with structure closely resembling that of Nb_3_TeI_7_, hosts an SOC-isolated flat band with the same band representation characteristics. Two further layer group 69 compounds, Ta_3_SBr_7_ (2dm-5348) and Ta_3_TeI_7_ (2dm-5496), likewise have Ta-dominated flat bands pinned within a few tens of milli–electron volts of the Fermi level, generated by their breathing kagome Ta sublattices. Complete band structures and atomic configurations for Ta_3_SBr_7_ are provided in Fig. 5G.

Within the C2DB database, Cu_3_AsO_4_ and Cu_3_SbO_4_ emerge as two structurally similar systems with nontrivial flat-band features. Both materials belong to layer group 69 (P3m1). The irreducible representations at HSPs Γ, *K*, and *M* in the momentum space are calculated and compared with irreps of EBRs. As shown in Fig. 5 (E and F), the third highest valence band lies close to the Fermi level and is spectrally isolated due to SOC. The decomposition of band representations is $\Gamma_{4}⊕\Gamma_{5}$, $K_{5}⊕K_{6}$, and $M_{3}⊕M_{4}$, which corresponds to $\left[{1c|}^{1}{\bar{E}}^{2}\bar{E}↑G\left(2\right)\right]⊕\left[1c|{\bar{E}}_{1}↑G(2)\right]⊖\left[1b|{\bar{E}}_{1}↑G(2)\right]$. Through summation of representations of this topological band and the two bands below that have trivial topologies, a linear combination of irreps of EBRs can be built, suggesting the fragility of the nontrivial topology. These features mirror the topological flat bands found in kagome systems, reinforcing the capability of our framework in uncovering topologically nontrivial flat-band candidates from both DFT-labeled (2DMatPedia) and out-of-distribution (C2DB) structural datasets.

## DISCUSSION

Our structure-informed framework represents a methodological advance in quantum materials screening by combining interpretable supervision with structure-property representation learning. The physics-motivated flatness score provides a transparent and physically grounded training signal, while the multimodal encoder captures latent structure-property relationships directly from atomic structure inputs. UMAP projections of the learned latent space reveal a clear separation between low- and high-flatness materials, with kagome-like motifs clustering in distinct regions—consistent with their known role in flat-band formation. When embeddings from both training (2DMatPedia) and inference (C2DB) datasets are projected into the same space, the separation by flatness score is preserved. While previously unseen structural motifs emerge in the C2DB domain, the predicted score distribution remains stable with only localized deviations, indicating that the model captures transferable geometric features rather than dataset-specific correlations.

When applied to unseen 2D materials (C2DB), we identified 55 kagome-like candidates with high predicted flatness, all found directly from atomic structure. DFT calculations were performed to obtain band structures for the screened candidates, and flatness scores were then reevaluated using the same physics-based metric defined during training. Among the 55 predicted high-flatness materials, 98.2% retained DFT-derived scores above 0.9, confirming the predictive accuracy of the framework.

The framework uncovered several previously unreported 2D materials hosting isolated flat bands with fragile topological character, unified by kagome-like sublattice motifs and symmetry features associated with nontrivial band topology. These materials share breathing kagome sublattices and belong to layer group 69 (P3m1), emphasizing the geometric motifs underpinning flat-band formation. In particular, Nb_3_TeI_7_, Ta_3_SeI_7_, Ta_3_SBr_7_, Ta_3_TeI_7_, Cu_3_AsO_4_, and Cu_3_SbO_4_ all exhibit spin orbit–induced band isolation for the first degenerate bands below their Fermi levels and representations of the symmetry group inconsistent with EBR, indicating nontrivial topology. Their discovery, enabled solely by structure-based screening and confirmed by DFT calculations, highlights the effectiveness of our flatness score and the model’s ability to capture hidden structure-property relations linked to flat-band behavior.

In summary, we present a framework that enables the direct screening of 2D materials from atomic structure without requiring electronic structure inputs. It is specifically designed for large-scale discovery of 2D flat-band materials that integrates a physics-informed flatness score with multimodal graph and language models. This approach leads to the identification of a collection of kagome-like flat-band materials, including both known and previously unrecognized candidates with DFT-confirmed fragile topological flat bands. While this study focused on flat-band materials discovery, the approach is readily extensible. By modifying the target scoring function, for example, to optimize exchange splittings or symmetry indicators, the same architecture could accelerate the search for 2D magnets, spin-liquid platforms, or other quantum phases. Its generalizability also supports application to 3D layered compounds and van der Waals heterostructures using only atomic inputs. While the current sublattice filter focuses on kagome motifs, future extensions could broaden motif coverage and incorporate symmetry-aware architectures to capture a wider range of flat-band geometries and related emergent phenomena.

## MATERIALS AND METHODS

### Flatness scoring

To systematically identify flat bands from the band structures of reported 2D materials, we develop a physically motivated flatness evaluation method. This approach quantifies the energy dispersion of bands across the Brillouin zone, augmented by the scores of DOS peaks to capture the concentration of electronic states and their proximity to the Fermi level, providing a foundation for subsequent machine learning predictions.

#### Band selection and Fermi level relevance

Flat bands are physically most important when located near the Fermi level, as they directly influence the low-energy physics of the material. Accordingly, we select a subset of bands from the material’s band structure that are closest in energy to the Fermi level. Specifically, for a given 2D material, we compute the average energy of each band and rank them based on their energy difference from the Fermi level, choosing the *n* bands with the smallest differences (or all bands if fewer than six are available). For spin-polarized materials, spin-up and spin-down channels are evaluated separately to capture spin-dependent features relevant to magnetic or strongly spin orbit–coupled systems.

To capture their dispersion characteristics, we examine these bands along continuous *k*-point paths in the Brillouin zone, constructed by connecting high-symmetry points. Each selected band is further segmented into continuous, noncrossing segments by identifying crossings with adjacent bands at each *k*-point. These segments are then reconnected at crossing points to form multiple full dispersion curves along high-symmetry paths. The curve with the narrowest energy span is then identified as the representative band for flatness evaluation of the material.

#### Definition of flatness score

We define a composite score $S_{\text{total}}$ that integrates $S_{\text{bandwidth}}$ and $S_{\text{DOS}}$, emphasizing minimal dispersion and high state density near the Fermi level. The bandwidth component, $S_{\text{bandwidth}}$, directly quantifies the flatness of the representative band as follows

$$S_{\text{bandwidth}}=\left\{ \begin{array}{c} -1,\;\text{if}\;\Delta E>\omega_{\text{max}}\\ \frac{1}{2}\left[\text{cos}\left(\pi \cdot \Delta E/\omega_{\text{max}}\right)+1\right],\;\text{otherwise} \end{array}\right.$$ (1)

Here, $\omega_{\text{max}}$ is a tunable threshold that sets the maximum bandwidth still considered “flat.” Bands with energy spans exceeding this threshold automatically receive zero scores. To accommodate diverse materials and balance physical selectivity with data coverage, we consider multiple values of $\omega_{\text{max}}$ (100, 300, and 500 meV) in our experiments. The cosine transformation ensures that the score is maximized ($S_{\text{bandwidth}}=1$) for a perfectly flat band and decreases smoothly to zero as the bandwidth approaches $\omega_{\text{max}}$. This nonlinear decay emphasizes sensitivity to small variations in bandwidth at low values, consistent with the physical principle that strong correlation effects emerge when electronic kinetic energy is much smaller than the interaction energy. Bands with large dispersion rapidly lose relevance for correlated phenomena and are assigned a low or zero score.

The DOS component, $S_{\text{DOS}}$, quantifies the concentration and peak prominence of electronic states within the representative band’s energy range. We measure how concentrated the DOS is around the band’s midpoint energy. The average DOS score of the local window and the broader surrounding DOS average within a fixed reference range of [−5 eV, 5 eV] (*25*) are computed as follows

$${\text{DOS}}_{\text{avg}}=\frac{1}{N}\sum_{i=1}^{N}\text{DOS}\left(E_{i}\right),\;{\text{DOS}}_{\text{all}}=\frac{1}{M}\sum_{j=1}^{M}\text{DOS}\left(E_{j}\right)$$ (2)

with $E_{i}$ sampled in the local window, $E_{i}\in \left[E_{\text{mid}}-\frac{1}{2}\omega_{\text{max}},E_{\text{mid}}+\frac{1}{2}\omega_{\text{max}}\right]$, and $E_{j}$ in a broad reference window of [−5,5] eV, $E_{j}\in \left[-5\;\text{eV},5\;\text{eV}\right]$. The final score $S_{\text{DOS}}$ is then calculated as follows

$$S_{\text{DOS}}=\frac{{\text{peak}}_{\text{contrast}}}{1+{\text{peak}}_{\text{contrast}}},\;\text{where}\;{\text{peak}}_{\text{contrast}}=\frac{{\text{DOS}}_{\text{avg}}}{{\text{DOS}}_{\text{all}}}$$ (3)

This formulation constrains $S_{\text{DOS}}$ to the range [0, 1], yielding a score of 0 when the local DOS is indistinguishable from the background average, and approaching 1 as a sharp peak emerges near the Fermi level. This provides a simple yet physically grounded indicator of localized electronic states that may enhance interaction-driven phenomena in flat-band systems.

The total flatness score combines the above components through a sigmoidal weighting scheme

$$\begin{array}{c} S_{\text{total}}=\left\{\begin{array}{c} 0,\;\text{if}\;\Delta E>\omega_{\text{max}}\\ \text{sigmoid}\left[\lambda \cdot \left(S_{\text{bandwidth}}+S_{\text{DOS}}\right)\right]\cdot \end{array}\right.\\ \text{sigmoid}\left[\beta \cdot \left(S_{\text{bandwidth}}\cdot S_{\text{DOS}}\right)\right],\;\text{otherwise} \end{array}$$ (4)

The first term rewards additive contributions, while the second accentuates cases where both dispersion suppression and DOS concentration co-occur. Parameters λ and β are tuned via BO (*48*), using a target function shaped by HDBSCAN clustering (*49*). The optimization objective linearly combines three terms: (i) the mean $S_{\text{total}}$ within the high-density cluster (or 0 if absent), promoting high scores in structurally relevant regions; (ii) the mean $S_{\text{total}}$ in low-score regions (where either input falls below the 10th percentile), penalizing undesired elevation; and (iii) the fraction of samples with $S_{\text{total}}>0.95$, to prevent score saturation. This formulation balances local fidelity with global regularization, encouraging physically meaningful score distributions. The final score is normalized to [0,1] and used as a regression target. Additional details of the flatness scoring formulation are provided in the Supplementary Text (section A).

### Details of the deep learning model

#### Data preprocessing

To construct a multimodal representation of 2D materials, we process data from 2DMatPedia and C2DB into graph and text modalities. Each entry provides crystal structure graph (the atomic species, lattice vectors, and atomic positions) and textural property features exploration.

To represent the atomic structure of 2D materials, we convert each crystal into a graph-based representation. Each structure is expanded along the *c* axis to suppress interlayer interactions under periodic boundary conditions

$$L_{c}^{\prime }=c_{\text{multi}}\cdot L_{c}$$ (5)

where $L_{c}$ is the original lattice vector along the c axis, and $c_{\text{multi}}$ is a scaling factor that ensures a sufficiently large separation between periodic images. The transformed structure is then used to compute interatomic distances and neighboring relationships.

From the modified geometry, an undirected atomistic graph G=(V,E) is constructed, where nodes correspond to atoms with CGCNN-derived elemental features, and edges represent interatomic bonds parameterized by interatomic distances. The *k* nearest neighbors are identified adaptively based on in-plane lattice constants. To encode local geometric environments beyond pairwise distances, we construct a line graph L(G), where each node corresponds to a bond in *G* and each edge encodes the angle between bond triplets

$$z_{\left(i,j\right)}=\text{cos}\left[\theta_{\left(i,j,k\right)}\right]$$ (6)

where $\theta_{\left(i,j,k\right)}$ represents the angle between three connected atoms. This dual-graph formulation captures both radial and angular dependencies, allowing the model to learn from local geometric constraints crucial for electronic behavior in low-dimensional materials.

In addition to structural graphs, we incorporate tabulated crystal attributes, summarized in Table 1, to provide complementary information. These include symmetry descriptors, lattice parameters, atomic species, and formula representations. Each property is converted into a structured natural language sentence using a fixed template, forming a sequence of material-specific descriptors. The resulting text is tokenized and encoded using a pretrained RoBERTa model (*41*), generating semantic embeddings for multimodal integration.

**Table 1.** Selected feature list. Each feature is accompanied by a specific description explaining its physical significance and contribution to material characterization.

Feature	Description
Formula (anonymous)	Generic chemical formula that removes specific element identifiers
Formula (pretty)	Human-readable chemical formula of the material
Space group	Space group indicating symmetry of the crystalline structure
Point group	Point group indicating symmetry around a point
Crystal system	Geometric classification of the crystalline structure
Lattice matrix	Matrix representation of the lattice
Lattice parameters	Lattice constants and angles
Atomic species	Species of atoms in a unit cell
Fractional coordinates	The positions of atoms in the unit cell in fractional coordinates
Lattice volume	Volume of the unit cell based on lattice parameters

#### Model architecture

We propose a deep learning model that integrates atomic structures, textual property descriptions, and band-related features for predicting the flatness of electronic bands in 2D materials. The model combines three primary components: a structure-aware graph encoder, a semantic-aware text encoder, and a bilinear interaction module to effectively fuse information from different modalities.

Our graph encoder is inspired by the ALIGNN framework (*40*) with a customized modular architecture for 2D materials to capture both bond-level and angular interactions through coupled graph and line graph representations. In the first stage, Line-GNN layers update angle features *z* and bond features *y* by propagating information on the line graph L(G), where each node represents a bond in the original atomic graph *G*. These updates incorporate angular dependencies among neighboring bonds. Simultaneously, atomic features *x* are updated through their interactions with bonds

$$x^{\prime }=x+F\;\text{node}\left(x,y\right),y^{\prime }=y+F\;\text{edge}\left(y,z\right)$$ (7)

where *x*, *y*, and *z* denote atomic, bond, and angle features, respectively. This triplet-aware mechanism allows the model to encode higher-order geometric correlations essential to 2D material behavior. In the second stage, Gate GNN layers further refine node and bond embeddings via edge-gated convolutions. Bond features are updated using triplet-based angular information

$$y_{\mathrm{ij}}^{\left(l+1\right)}=y_{\mathrm{ij}}^{\left(l\right)}+\text{MLP}\left[\sum_{k\in N\left(ij\right)}\sigma_{ijk}\cdot z_{ijk}\right]$$ (8)

Updated bond embeddings guide the message passing among atoms, producing refined atomic features $x^{\left(l+1\right)}$. Last, the graph-level representation is obtained by average pooling over atomic nodes

$$h_{\text{out}}=\frac{1}{|V|}\sum_{i\in V}x_{i}^{\left(\text{final}\right)}$$ (9)

This embedding serves as the structural input to the multimodal prediction module.

For the property representations, the textual input $T_{\text{info}}$ is first tokenized and passed through a pretrained RoBERTa model. The final embedding is extracted from the [CLS] token, representing the overall material description. This embedding is further projected through a linear layer with tanh activation and an MLP

$$z_{T}=\text{MLP}\left(\text{tanh}\left(W_{\text{lin}}\cdot \text{RoBERTa}\left([\text{CLS}]\right)\right)\right)$$ (10)

This yields a compact semantic representation $z_{T}\in {\mathbb{R}}^{d}$ that encodes global text-derived properties. To fuse the structural and semantic modalities, we first project both embeddings into a shared latent space and then apply a bilinear attention mechanism between the graph encoder output $h_{\text{out}}$ and the text embedding $z_{T}$. The joint representation is computed as

$$z_{F}=h_{G}^{⊤}{\mathrm{Wz}}_{T}$$ (11)

where $W\in {\mathbb{R}}^{d\times d\times d}$ is a learnable weight tensor. This interaction captures high-order correlations between atomic geometry and descriptive semantics. The fused vector $z_{F}$ is passed through a multilayer perceptron to predict a scalar flatness score. This end-to-end architecture enables joint reasoning over atomic geometry and material semantics, providing accurate predictions for flat-band characteristics in 2D materials.

#### Training and evaluation

To robustly assess the performance of our model, we used a 5-fold cross-validation strategy. In this setting, the dataset is partitioned into five subsets of approximately equal size. In each fold, 80% of the data are used for training and 20% are used for validation. We use the Adam optimizer with a scheduled learning rate reduction based on validation loss plateauing. For each epoch, model parameters are updated based on the mean square error (MSE) between predicted and true flatness scores. Gradients are clipped to prevent explosion, and the model with the best validation *R*^2^ score in each fold is saved for further analysis.

The model’s performance is evaluated with a suite of regression metrics, including RMSE, coefficient of determination (*R*^2^), and MSE. The primary metric, RMSE, measures the average magnitude of prediction errors and is defined as

$$\text{RMSE}=\sqrt{\frac{1}{n}\sum_{i=1}^{n}{\left(y_{ie}-y_{ip}\right)}^{2}}$$ (12)

where $y_{ip}$ and $y_{ie}$ represent the predicted and experimental flatness scores, respectively, and *n* is the number of samples in the validation or test set. A smaller RMSE indicates that the predicted values are close to the actual values. In addition, the *R*^2^ metric assesses how well the model explains the variability of the response variable

$$R^{2}=1-\frac{\sum_{i=1}^{n}{\left(y_{ie}-y_{ip}\right)}^{2}}{\sum_{i=1}^{n}{\left(y_{ie}-\bar{y}\right)}^{2}}$$ (13)

where $\bar{y}$ is the mean of the observed flatness scores. An *R*^2^ value closer to 1 indicates a better fit of the model to the data.

All predictions and evaluations are performed on unseen validation data in each fold, and the final performance is reported as the average across the five folds. This protocol ensures robust assessment of model performance and generalization.

#### Experimental setting

The deep learning model is implemented in Python 3.9, using PyTorch 2.6.0 (*50*) for neural network construction, DGL 1.1.0 (*51*) for handling lattice graph structures, scikit-learn 1.6.1 (*52*) for data preprocessing, pymatgen 2024.8.9 (*53*) for generating and analyzing 2D crystal structures, SciPy 1.13.1 (*54*) for numerical optimization, and transformers 4.48.3 (*55*) for implementing the RoBERTa model to extract serialized crystal structure features.

For training, the batch size is set to be 8 and the Adam optimizer is used with a learning rate of 4 × 10^−1^. We configured the model to use fivefold cross-validation, with each fold running for a maximum of 100 epochs. For each iteration, the model’s performance was monitored at every epoch on the validation set, tracking metrics MSE, RMSE, and *R*^2^. The best epoch within each iteration, determined by the highest *R*^2^, was selected. The final performance of the models was then calculated by averaging the metrics across all five iterations. This approach ensures that the evaluation captures the variability of the model’s predictions, providing a reliable estimate of its performance. The configuration details and ablation analysis are provided in the Supplementary Text (section B).

### Computational simulation

To further prioritize candidates likely to exhibit kagome-induced flat bands, we applied a geometric filtering step that identifies kagome-like sublattice motifs based on coplanarity, bond length ratios, and local angular symmetry. Full algorithmic details are provided in the Supplementary Text (section C).

We performed DFT simulation using the Vienna Ab Initio Simulation Package to calculate electronic properties including band structures and Kohn-Sham wave functions. We used the projector augmented wave (PAW) method and the Perdew-Burke-Ernzerhof exchange-correlation functional, similar to the Materials Project (*56*, *57*). To prepare input files and plot band structures after DFT simulation, the Pymatgen codebase is used (*53*, *57*). The plane-wave basis set had an energy cutoff of 520 eV. Structural relaxations were carried out until the forces on all atoms were smaller than 0.01 eV/Å, with an electronic convergence threshold of 10^−5^ eV. We used a Monkhorst-Pack 9 × 9 × 1 k-point mesh for geometry optimizations and self-consistent run, which was increased to 12 × 12 × 1 for DOS evaluation. The band structure computation uses line-mode k-point grid along the HSPs of the 2D Brillouin zone. Van der Waals interactions were accounted for using the dispersion-corrected vdW-optB88 exchange-correlation functional. A vacuum gap of at least 20 Å was maintained along the *c* axis following the same standard of 2dMatPedia and C2DB. All structures were fully relaxed (ISIF = 3) while maintaining the fixed vacuum gap. To compute irreducible representations, we used the protocol described in (*58*) using “irvsp” to calculate irreps from wave functions and “phonopy” (*59*, *60*) and “pos2aBR” (*61*, *62*) to prepare standard POSCAR files. The Bilbao Crystallographic Server (*63*–*65*) is used to compare band representations with irreps of EBRs of layer groups (*44*). To draw band structures from simulation with SOC, the “pyprocar” codebase (*66*, *67*) is used.

## Supplementary Materials

### This PDF file includes:

Supplementary Text Figs. S1 to S8 Tables S1 and S2 References
