# Supplementary material for: Discovery of flat-band 2D materials via physics-informed scoring and structure-based learning
Source: Sci Adv. 2026 Jul 8;12(28):eaea3611. doi: 10.1126/sciadv.aea3611 (PMC13344314; doi:10.1126/sciadv.aea3611)
Supplement: Supplementary file 1 — Supplementary Text Figs. S1 to S8 Tables S1 and S2 References [file sciadv.aea3611_sm.pdf]

Science Advances
AAAS

Supplementary Materials for

Discovery of flat-band 2D materials via physics-informed scoring and structure-based learning

Xiangwen Wang *et al.*

Corresponding author: Xiangwen Wang, xiangwen.wang@manchester.ac.uk; Qian Yang, qian.yang@manchester.ac.uk; Artem Mishchenko, artem.mishchenko@manchester.ac.uk

Sci. Adv. **12**, eaea3611 (2026)
DOI: 10.1126/sciadv.aea3611

This PDF file includes:

Supplementary Text
Figs. S1 to S8
Tables S1 and S2
References

Supplementary Text

A Flatness score optimization

A.1 Effect of Bandwidth Threshold on Flatness Scoring

This section evaluates how different bandwidth thresholds (ω_{max} = 0.1, 0.3, 0.5 eV) influence the shape and outcome of the flatness scoring function. As shown in Figure S1, for each setting, a Bayesian optimization process was conducted over the scoring parameters (λ, β), as shown in the left column, to maximize the contrast between flat and non-flat bands. The middle column presents the distributions of final flatness scores (S_{total}) using the best parameters, while the right column shows the correlation between the two score components ($S_{bandwidth}$ and S_{DOS}).

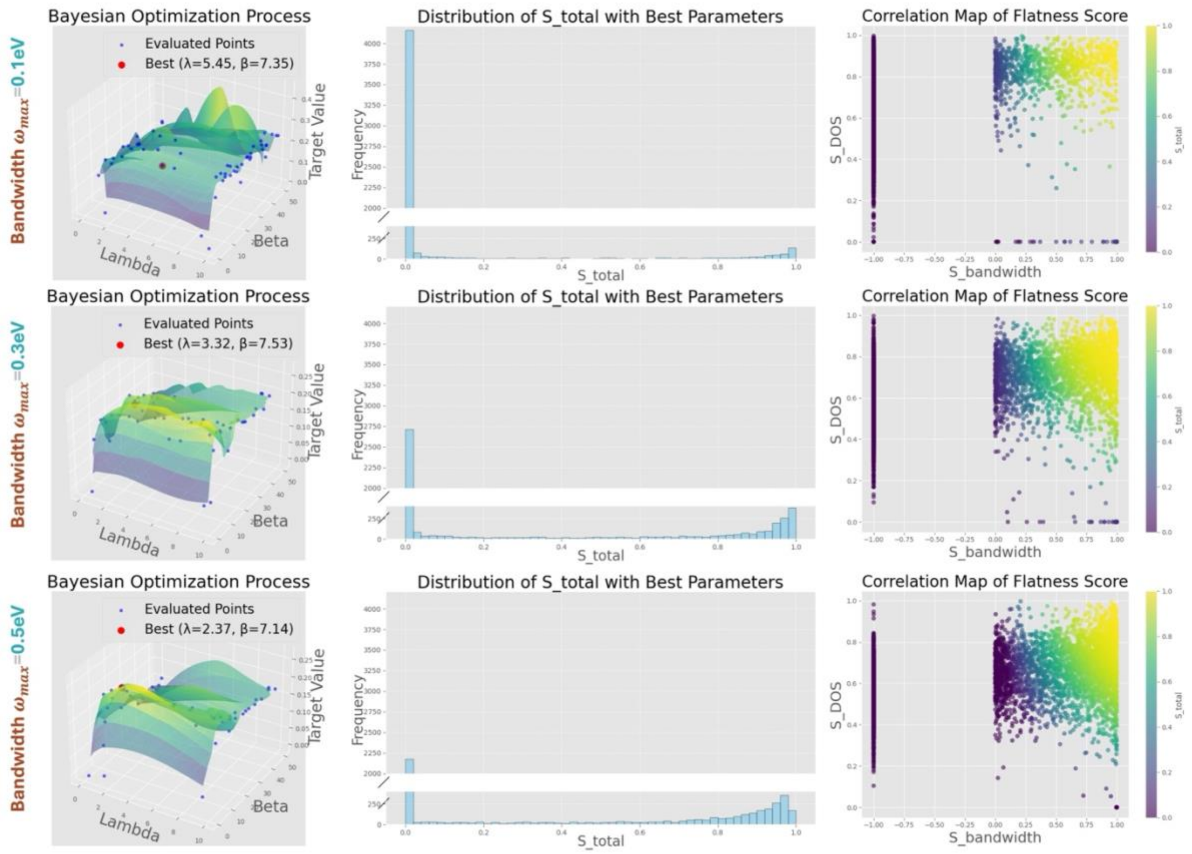

Fig. S1 Effect of bandwidth threshold ω_{max} on flatness scoring. Left: Bayesian optimization surfaces and selected optimal parameters (λ, β) for each ω_{max}. Middle: Histogram of final S_{total} distribution using best parameters. Right: Scatter plot of $S_{bandwidth}$ and S_{DOS}, colored by S_{total}, showing how the composite score reflects the underlying component relationship at each threshold.

At low ω_{max} = 0.1 eV, scoring yields a sharp dichotomy with little resolution across moderate band structures. In contrast, ω_{max} = 0.5 eV introduces overly broad bandwidth tolerance, making score differentiation less selective. The intermediate setting ω_{max} = 0.3 eV balances sensitivity and selectivity, producing a well-distributed S_{total} and clear score stratification across the structural space. Notably, it also yields a sufficient number of high-scoring entries near 1, rather than concentrating most scores at 0, resulting in a more balanced distribution. Based on this trade-off, we selected 0.3 eV as the final threshold for all main results.

A.2 Bayesian Optimization Procedure for Flatness Scoring

This section presents the pseudocode for the Bayesian optimization algorithm used to determine the optimal flatness scoring parameters. The algorithm, shown in Figure S2, searches over the weight parameters λ and β, as well as the minimum cluster size used in density-based clustering, to enhance score separation between high- and low-flatness regions. At each iteration, candidate scores are computed from combinations of $S_{bandwidth}$ and S_{DOS}, and a target value is derived based on the clustering characteristics of high-scoring entries. The optimization objective is designed to promote score distributions that simultaneously yield distinct high-density flat-band clusters and sufficient contrast across the dataset. The final scoring parameters are selected to maximize the acquisition value over all evaluations and are used to compute the optimized score S_{total}^{*}.

Algorithm Bayesian Optimization for Flatness Score S_{total}

Input: Dataset $\mathcal{D} = \{(S_{\text{bandwidth}}, S_{\text{DOS}})\}$, optimization budget T
Define search bounds for parameters: $\lambda \in [0, 10]$, $\beta \in [0, 10]$, min_cluster_size $\in [5, 10]$
Initialize Bayesian optimizer with Gaussian Process surrogate model
for $t = 1$ **to** T **do**
    Select next parameters using acquisition function:
        $(\lambda_t, \beta_t, \text{min\_cluster\_size}_t) \leftarrow \arg\max \mathcal{A}(\cdot|\mathcal{D}_{t-1})$
    Compute S_{total} using:
        $S_{\text{total}} = \sigma(\lambda_t(S_{\text{bandwidth}} + S_{\text{DOS}})) \cdot \sigma(\beta_t(S_{\text{bandwidth}} \cdot S_{\text{DOS}}))$
    Apply HDBSCAN clustering on squared descriptors
    Identify high-density cluster closest to $(1, 1)$
    Compute target score:
        $y_t = w_1 \cdot \mu_{\text{high}} - w_2 \cdot \mu_{\text{low}} - w_3 \cdot \text{ratio}_{\text{high-}S_{\text{total}}}$
    Append result to dataset: $\mathcal{D}_t \leftarrow \mathcal{D}_{t-1} \cup \{((\lambda_t, \beta_t, \text{min\_cluster\_size}_t), y_t)\}$
end for
Select best parameters:
    $(\lambda^*, \beta^*, \text{min\_cluster\_size}^*) = \arg\max_{(\cdot)} \mathcal{D}_T$
Recompute normalized S_{total} using best parameters
Set $S_{\text{total}} = 0$ for invalid entries ($S_{\text{bandwidth}} = -1$)
Output: Optimized flatness score S^*_{total} and best parameters

Fig. S2 Pseudocode for the Bayesian optimization algorithm used to tune flatness score S_{total}. The procedure iteratively updates scoring parameters (λ, β) and clustering settings to promote separable and meaningful distributions of flatness scores. The final optimized score is computed based on the best-performing parameter set.

B Predictive Deep learning model

B.1 Hyperparameter optimization and training dynamics.

Table 1 summarizes the key hyperparameters explored for each model component, including GNN, BERT, BAN, and training strategy, along with their candidate values and functional descriptions. Corresponding training and validation performance curves under different settings are shown in Figure S3, evaluated on a representative subset of the data. These comparisons provide empirical support for the selected configuration, highlighting its stability and convergence behavior across model variants.

Table S1 Summary of hyperparameter settings.

Module	Parameter	Options	Description
**GNN**	Hidden feature dimension	[64, 128, **256**]	Feature dimension of each GNN layer
**Full-Alignn**	Line-GNN layers / GCN layers	[4/4, **4/2**, 2/4, 3/3]	Number of layers for global topology and local geometry modelling
**BERT**	Hidden layers	[8, **12**, 16]	Number of hidden Transformer layers
**BAN**	Bilinear feature dimension	[64, **128**, 256]	Dimensionality for bilinear interaction between graph and context embeddings
**Training**	Learning rate	[0.001, 0.0005, **0.0001**]	Learning rate for optimization
	Batch size	[**8**, 16]	Size of each training batch

Bolded values indicate the selected configuration used in the final model, chosen based on validation performance on a representative data subset.

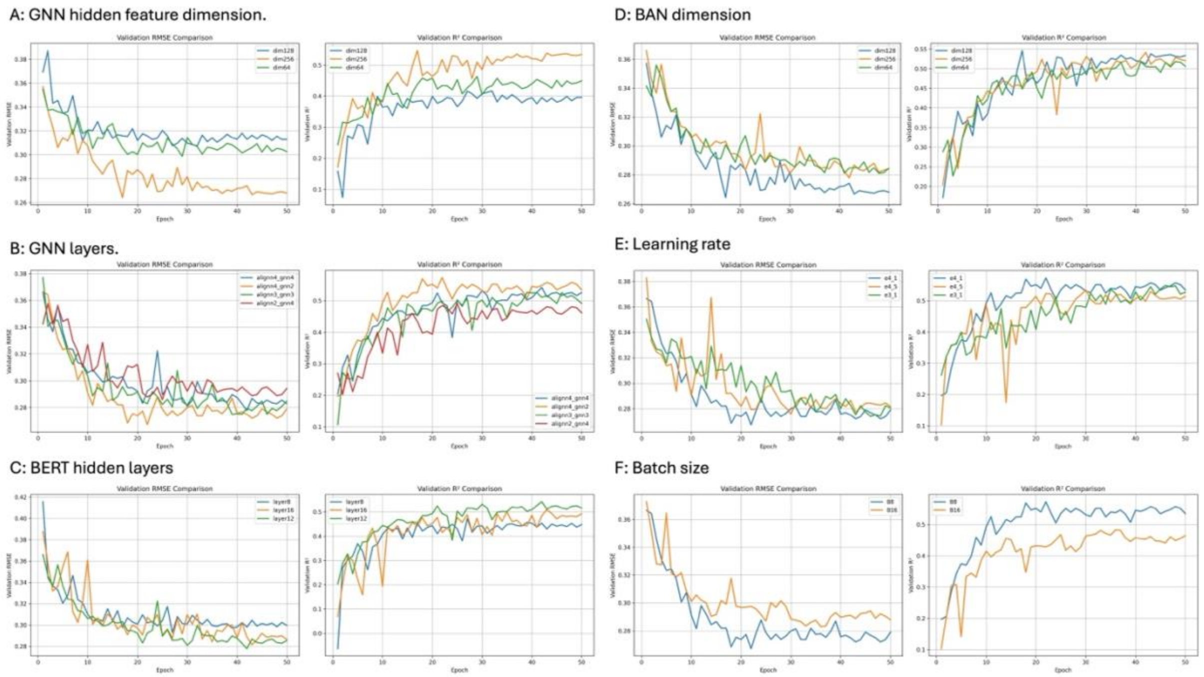

Fig. S3 Validation performance under different hyperparameter configurations. Validation RMSE (left panels) and R^2 score (right panels) for different settings of key hyperparameters: A. GNN hidden feature dimension, B. GNN layer configuration, C. BERT hidden layers, D. BAN feature dimension, E. learning rate, and F. batch size. Results are evaluated on a representative subset of the dataset.

B.2 Model Calibration

To further interpret the calibration behaviors, we analyze the predicted and target flatness scores across different score regimes, shown in Figure S4.

At low predicted scores, the model exhibits a systematic underestimation relative to the Bayesian-optimized target values. This regime is characterized by a large population of materials with weak or negligible flat-band features, for which the composite flatness score is dominated by low bandwidth and DOS contributions. The observed behavior is consistent with a conservative screening bias, whereby the model avoids assigning elevated scores to low-quality candidates.

In the intermediate score range (between 0.3 to 0.6), the deviations between predicted and target values are moderate, typically on the order of 0.05--0.07. Materials in this regime correspond to borderline cases with partial flat-band characteristics and are not directly selected by score thresholding but instead require additional motif-based filtering and first-principles validation.

In contrast, in the high-score regime relevant for candidate discovery (higher than 0.8), the predicted scores closely follow the target values, with deviations below 0.01 in the highest-score bins. This region shows both good calibration and substantial sample counts, indicating that the agreement is statistically robust. The strong alignment between predicted and target scores in this regime confirms that the model faithfully reproduces the Bayesian-optimized composite flatness score where accurate ranking is most critical for large-scale screening.

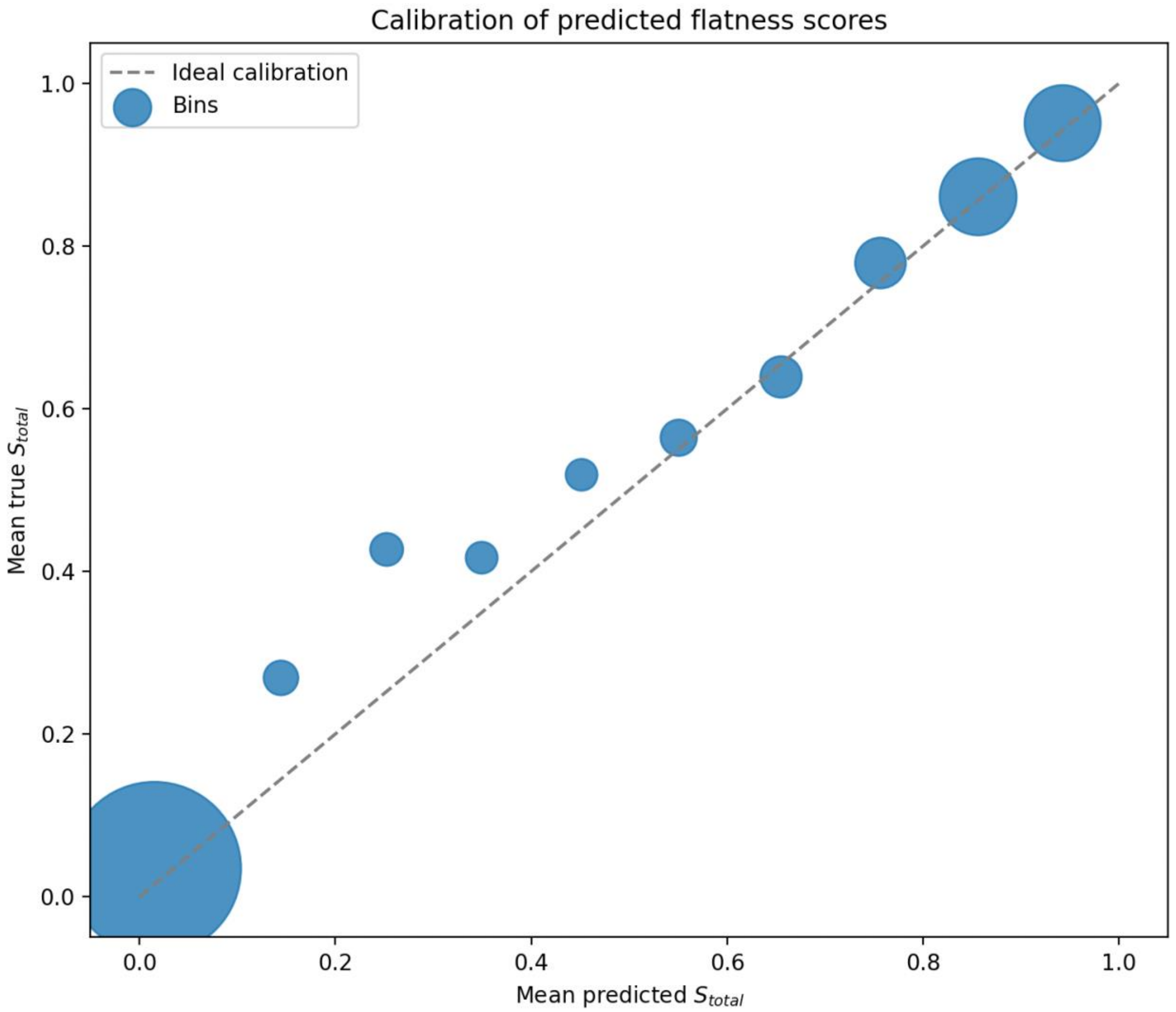

Fig. S4 Calibration of predicted composite flatness score. The analysis is performed on the same 2DMatPedia dataset used for model training and evaluation in Fig. 3c. Predicted scores are binned and compared with the corresponding Bayesian-optimized target flatness scores. Marker size reflects the number of samples in each bin, and the dashed line indicates ideal calibration.

B.3 Model Interpretation via Structural Embedding Analysis

This section presents an interpretation of the model by analyzing the structural embeddings. A UMAP clustering analysis are applied to the learned embeddings to reveal patterns and groupings associated with model behavior and prediction outcomes.

Cluster-level analysis was performed to assess differences in flatness score, structural similarity, and sample distribution. As shown in the left panel of Figure S5, clusters 2 and 3 exhibit markedly higher mean flatness scores with larger variance, indicating that high-scoring structures tend to concentrate in these regions of the latent space. In contrast, other clusters (e.g., 0, 1, 4–6) show significantly lower flatness values. The middle panel presents intra-cluster similarity metrics, where cosine similarity remains consistently high across clusters, but Euclidean distance and compactness show notable variability, suggesting different degrees of internal cohesion. The right panel indicates an approximately balanced distribution of points across clusters, with clusters 2 and 3 containing the most samples. These observations reinforce that the UMAP-based embedding space captures meaningful structural variation aligned with flat-band characteristics.

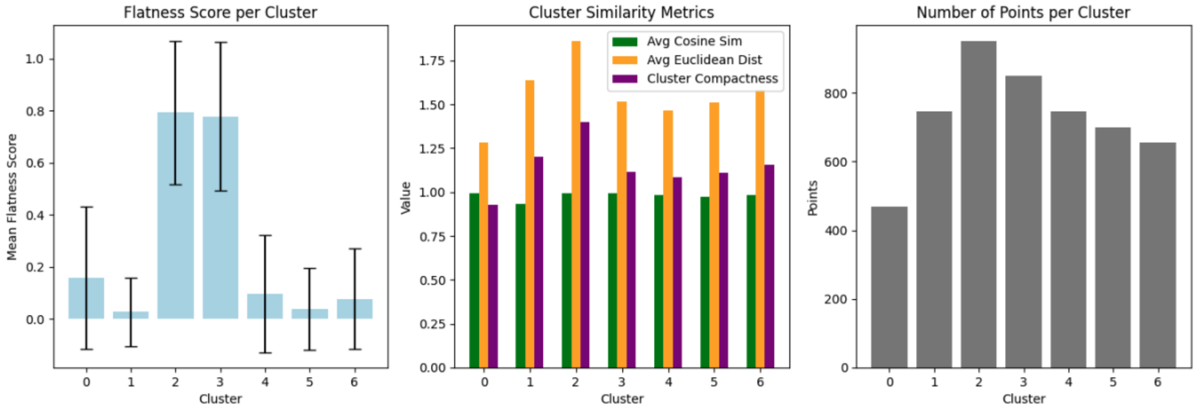

Fig. S5 Analysis of latent space clusters after selecting n=7. Left: Mean flatness score with standard deviation for each cluster. Middle: Cluster similarity metrics including average cosine similarity (green), average Euclidean distance (orange), and cluster compactness (purple). Right: Number of samples assigned to each cluster.

To assess the robustness and out-of-distribution (OOD) generalization of the learned representations beyond random train–test splits, we performed a global leave-one-cluster-out (LOCO) evaluation following recent best practices in extrapolative testing for materials machine learning [68]. Unlike random splits, which primarily probe interpolation within the training distribution, LOCO evaluation explicitly tests extrapolation across latent structural regimes by holding out entire clusters of samples during training.

Latent embeddings were first extracted from the trained GNN encoder for all samples in the dataset. Importantly, the GNN model was not retrained for this analysis. The embeddings were standardized and clustered using k-means clustering with k=7, corresponding to the number of visually distinct regions observed in the latent space projections. Clustering was performed in an unsupervised manner and did not use any target property information, ensuring that the resulting clusters reflect intrinsic structural similarity as learned by the model.

For LOCO evaluation, each latent cluster was iteratively treated as a held-out test set, while all remaining clusters were used for training. For each split, training set contains all samples outside the selected cluster; test set contains all samples within the held-out cluster. To isolate the generalization properties of the learned representations, a simple ridge regression model with standardized inputs was trained on the latent embeddings of the training set and evaluated on the held-out cluster. This choice avoids confounding the analysis with additional model capacity or retraining effects and directly probes the transferability of the learned embedding space. For comparison, a random-split baseline was constructed using 20 independent random train–test splits with the same test fraction as a single LOCO fold (around 1/7 of the dataset).

The results of the global LOCO evaluation are summarized in Figure S6, which reports overall performance degradation relative to random splits, cluster-wise prediction errors, and the corresponding cluster size distribution. LOCO evaluation leads to a clear degradation in both MAE and RMSE compared to the random-split baseline. This degradation is expected for a genuine OOD setting, as entire latent clusters are excluded from training. Importantly, while the error increase is substantial, it is non-catastrophic, indicating that the learned representation retains meaningful predictive capability even under this stringent evaluation.

To further examine heterogeneity across latent regimes, middle panel reports cluster-wise MAE and RMSE for each LOCO fold. Performance varies across clusters, reflecting differences in extrapolation difficulty between structural regimes; however, no single cluster dominates the overall degradation. This suggests that the observed performance drop is a general consequence of out-of-cluster prediction rather than an artifact of a small or pathological subset of the data. The right panel shows the number of samples assigned to each latent cluster. All clusters contain comparable numbers of samples, confirming that the LOCO results are not driven by cluster size imbalance and that each held-out evaluation reflects a statistically meaningful test set.

Taken together, these results demonstrate that the learned latent space exhibits well-defined structural regimes, that extrapolation across these regimes is significantly more challenging than interpolation within them, and that the resulting performance degradation under LOCO evaluation is consistent with prior observations in extrapolative materials machine learning studies.

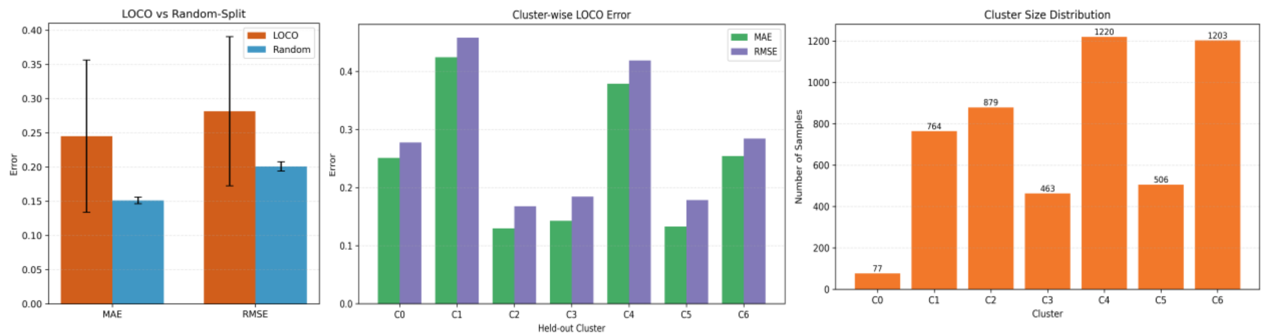

Fig. S6 Global LOCO evaluation of out-of-distribution generalization. Left: Comparison of MAE and RMSE between LOCO evaluation and random-split baselines, reported as mean standard deviation across clusters or random splits. Middle: Cluster-wise LOCO prediction errors (MAE and RMSE) for each held-out latent cluster, illustrating variability in extrapolation difficulty across structural regimes. Right: Distribution of sample counts across latent clusters used in the LOCO evaluation.

C Theoretical and Computational Validation

C.1 Sublattice Identification Methods

To determine whether a given 2D crystal structure contains a kagome-type sublattice, we designed a two-stage geometric and symmetry-based identification criterion based purely on atomic coordinates and neighbor geometry.

In the first stage, the objective is to detect a group of four neighbouring atoms that are approximately coplanar with a central atom. For a given i, its neighboring atoms within a cutoff radius r are identified. Among these, all possible combinations of four neighbors are enumerated. For each combination, the coplanarity is tested by computing the distance of all five points (the four neighbors and the central atom) from the plane defined by three of the neighbor atoms. If both the fourth neighbor and the central atom lie within a small orthogonal distance from the fitted plane, denoted as δ_{plane}, the configuration is considered coplanar. To ensure structural uniqueness, it is additionally required that no other neighbor outside the selected four lies in the same plane within tolerance.

In the second stage, the goal is to verify whether the selected four neighbors form two mirror-related equilateral triangles with the central atom. The four atoms are partitioned into two disjoint pairs. For each pair, the bond lengths connecting the central atom to the pair members are computed and checked to be approximately equal within a relative tolerance δ_{length}. The angle between the two bond vectors is also required to be close to $60°$ within an absolute angular tolerance δ_{angle}. Furthermore, the two triangles must exhibit approximate C_2 symmetry: the bisectors of the two bond pairs should point in opposite directions. This is verified by calculating the angle between the two bisector vectors and requiring it to be close to $180°$ within a given angular threshold $\delta_{bisector}$.

A structure is labeled as containing a kagome sublattice if such a central atom and its associated four neighbors satisfying both conditions can be identified, and this local motif is repeated consistently among its neighbors. In many crystals, sublattice structures are only approximate Kagome and still host flat bands when the distortion is small, thus we set a generous tolerance for geometric features. The values used in our analysis are: cutoff radius $r = 6.5\text{Å}$, $\delta_{plane} = 0.1\text{Å}$, $\delta_{length} = 0.2$, $\delta_{angle} = 5°$ and $\delta_{bisector} = 5°$.

C.2 Kagome-like flat-band materials

This dataset contains 55 flat-band material candidates identified by our structure-informed model. The predictions were made on atomic structures from C2DB. Structures with predicted flatness scores ⩾ 0.9 were first selected, and kagome-like motifs were further filtered based on geometric criteria. DFT calculations were then performed on these filtered candidates to obtain band structures. The results confirm a high prediction accuracy, with 98.2% of the DFT-computed flatness scores remaining above 0.9.

Table S2. Dataset of kagome-like flat-band materials identified from C2DB structures.

Key	S_bandwidth	S_DOS	S_total
1BiHS2O6-1	0.7596	0.81194614	0.95895872
1GaNaSb2O6-1	0.9638	0.83618096	0.98750308
1SeNb3F7-1	0.9939	0.66062002	0.96914259
1InNaP2O6-1	0.8761	0.87364794	0.98394792
1SrAs2F12-1	0.9206	0.87428266	0.98736078
1AgGaSb2O6-1	0.7782	0.72995658	0.94368765
2AsFGeO3-1	0.9188	0.80953458	0.98173871
1STa3Br7-1	0.954	0.69425082	0.96992671
2TiF3-1	0.8588	0.79746001	0.9735003
2FGeSbO3-1	0.9422	0.85218156	0.98724096
1Bi2Sn3O9-1	1	0.81564994	0.98828175
1FeSn2C6N6-1	0.9692	0.7307688	0.97750145
1InNaSb2O6-1	0.9449	0.85586062	0.98767229
2PbSbO3-2	0.9563	0.88377696	0.99003928
1InLiAs2O6-1	0.9461	0.84346812	0.98687098
1NiSn2C6N6-1	0.9976	0.7236193	0.97946288
1NiPb2C6N6-1	0.9983	0.66554344	0.97065994
1SnK2F6O6-1	0.9972	0.89895767	0.99257087
1InNaAs2O6-1	0.9245	0.87527998	0.98768389
1TeNb3I7-1	0.9668	0.65769033	0.9645706
1SrSb2F12-1	0.9718	0.88115809	0.99067127
2GeSeO4-1	0.7029	0.77596067	0.93480289
1SnNa2F6O6-1	0.9859	0.82902033	0.98835874
1Nb3F8-1	0.9959	0.81109442	0.98771929
2As2O5-1	0.9793	0.90303441	0.99203807
1As2Sn3O9-1	0.9908	0.89456258	0.99213776
1TeTa3I7-1	0.9816	0.66276049	0.96784096
2ClGeSbO3-1	0.7972	0.8549563	0.97293748
1MgNa2S2F4O8-1	0.9622	0.86804218	0.98946142
2MoSbO5-1	0.7666	0.86531851	0.96965658
1MgLi2S2F4O8-1	0.9823	0.8527118	0.9896385

1MoSnO4-1	0.7152	0.85765893	0.95824318
1STa3I7-1	0.9547	0.69845731	0.97077052
1MnSn2C6N6-1	0.9431	0.75000108	0.9772386
1AsNiO3-1	0.7194	0.86097837	0.95983075
2ZnI2Sb2O3-1	0.9767	0.78580732	0.9843434
2ClSbSnO3-1	0.8404	0.88383399	0.98156276
1MnGe2C6N6-1	0.9824	0.81202318	0.98693467
1AlNaSb2O6-1	0.9509	0.86007891	0.98832486
1BeAs2F4O4-1	0.9838	0.91151459	0.99256982
1AlNaAs2O6-1	0.9085	0.87970807	0.98692068
1FSbS2O6-1	0.8536	0.75437211	0.96562807
1BaAs2F12-1	0.9401	0.87928994	0.98889456
1SNb3I7-1	0.8777	0.76364473	0.97099251
1SeNb3I7-1	0.9366	0.58635696	0.93796039
1GeNa2F6O6-1	0.9919	0.82484735	0.98843004
1GaNaAs2O6-1	0.9338	0.8754224	0.98827777
2CaBr2S2F4-1	0.9237	0.76748904	0.97738152
1BiFS2O6-1	0.9684	0.8025734	0.98520088
1SeTa3I7-1	0.97	0.61752433	0.95524689
1Sb2Sn3O9-1	0.9947	0.89927748	0.99248889
1FeGe2C6N6-1	0.9936	0.81857304	0.98810503
1BaSb2F12-1	0.9569	0.91002221	0.99139224
1BaBi2F12-1	0.9785	0.92439604	0.99287623

C.3 Statistical Validation and Model Performance

To rigorously evaluate the classification performance and exclude potential selection bias, we expanded the DFT validation set to include both positive and negative samples spanning the full predicted score range. Candidates from the C2DB database were divided into three groups according to their predicted flatness score: high (larger than 0.9), middle (0.3-0.9) and low (smaller than 0.3). In addition to the 55 previously validated high-scoring kagome-like candidates (Section SC.2), we randomly selected 20 materials from the middle-score subset and 20 materials from the low-score subset. These 40 materials were treated as negative validation samples and subjected to full DFT band-structure calculations under identical computational settings. The combined dataset (55 positive + 40 randomly sampled candidates) was used for further analysis in this section.

The corresponding precision-recall curve is presented in the left panel of Figure S7, while the confusion matrix, illustrating the distribution of correctly and erroneously classified samples, is shown in the right panel of Figure S7. The model demonstrated reliable classification performance, with a precision of 0.982 and a recall of 0.974. This indicates that 98.2% of predicted flat-band materials are confirmed by DFT; and 97.4% of true flat-band materials are successfully identified by the model. The normalized confusion matrix (Fig. S7B) summarizes: True Positives (TP), True Negatives (TN), False Positives (FP), False Negatives (FN). Both FP and FN rates remain low after inclusion of randomly sampled mid- and low-score candidates, confirming the robustness of the classification boundary.

To evaluate the screening efficiency, we calculated the enrichment factor (EF) defined as: (Precision in top x% ranked candidates) / (Baseline prevalence in dataset). For the top 20% highest-scoring candidates, $EF_{20\%}$ is 1.58. The baseline prevalence of flat-band materials in the pre-screened dataset is approximately $P \approx 0.6$. Therefore, random selection would yield a 60% success rate, while our model increases the precision to approximately 95% within the top-ranked subset. Although EF values are typically higher in highly imbalanced datasets, in this case the relatively high baseline prevalence constrains the maximum achievable EF. Under this condition, an $EF_{20\%}$ of 1.58 still represents a substantial gain in DFT screening efficiency, effectively reducing false-positive exploration.

These results demonstrate that the model performance remains stable and reliable when evaluated using randomly sampled mid- and low-score candidates, alleviating concerns regarding optimistic validation bias.

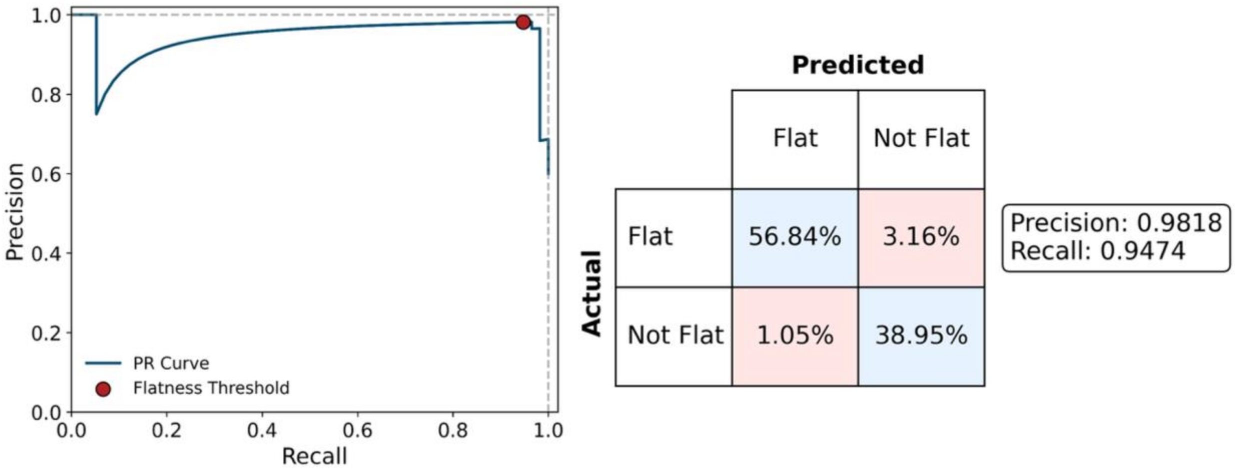

Fig. S7 Validation of model classification for flat-band materials. Left: The PR curve demonstrates high classification performance, with an AUC of 0.95. The red dot highlights the performance at the chosen flatness threshold of S_{total} = 0.9. (B) Confusion matrix showing the ratios of true positives, true negatives, false positives, and false negatives. The model achieves a high precision of 0.98 and a recall of 0.95, indicating strong reliability in identifying flat-band candidates.

C.4 Score Reliability

We systematically investigated the numerical robustness of the bandwidth score $S_{bandwidth}$ and DOS score S_{DOS}.

Robustness of the Bandwidth Score

The bandwidth score $S_{bandwidth}$ is defined as the minimum bandwidth of bands near the Fermi level and is calculated from energy eigenvalues along the high-symmetry lines (HSL) in the Brillouin zone (BZ). However, directly evaluating band flatness on a dense two-dimensional k-point mesh is challenging due to the band-crossing problem, i.e., the interchange of eigenvalue indices between neighboring k points. This mixing complicates the identification of a single continuous band across the BZ, a difficulty also discussed for the 1D case in the main text. Thus, we extended our 1D bandwidth-tracking algorithm to a continuous k-path that traverses the entire 2D k mesh. The path was constructed using a Hilbert space-filling curve, which connects all k points on a 36 × 36 mesh while preserving local continuity in k space.

Because the k points densely cover the entire 2D BZ, band crossings occur more frequently than along conventional HSL paths, increasing the complexity of tracking the flattest band. Nevertheless, $S_{bandwidth}$ was successfully computed for three representative materials: GeO_2 (2dm-2326), $LiCuCO_3$ (2dm-3825) and SiO_2 (2dm-3879). GeO_2 has an $S_{bandwidth}$ of 0.932 from BS on HSL and 0.929 on 2D k mesh. The $S_{bandwidth}$ of $LiCuCO_3$ is 0.980 and 0.986 from HSL and 2D BS respectively. And SiO_2 has $S_{bandwidth}$ of 0.954 and 0.956 from HSL and 2D BS. The values showed a deviation of less than 0.01 compared to the scores obtained from those calculated using HSL band structures. This high level of consistency confirms that the HSL-based bandwidth metric provides a numerically robust approximation of the full Brillouin-zone dispersion.

Robustness of the DOS Score

DOS calculations in DFT are sensitive to several numerical parameters, including the integration scheme, smearing width, and k-point density. To evaluate the robustness of the DOS score SDOS, we performed systematic parameter sweeps across nine randomly selected representative materials. The calculations were repeated using: Gaussian smearing widths of 0.01 eV, 0.05 eV, and 0.1 eV, the tetrahedron method, k-point meshes of 9×9×1 and 25×25×1. The fluctuations of S_{DOS} for nine representative, randomly sampled materials are illustrated in the top panel of Figure S8. The maximum deviation of S_{DOS} was 0.28. The largest variation occurs for HfP_2 (2dm-2394), where S_{DOS} ranges from 0.30 to 0.58. In general, fluctuations are more pronounced in the low-score regime, whereas S_{DOS} remains comparatively stable for high-scoring materials.

Importantly, these variations in S_{DOS} do not lead to significant changes in the total score S_{total}, which exhibits a maximum deviation of only 0.05 across all tested parameter combinations. No flat-band candidate changes classification due to these numerical variations. These results indicate that S_{DOS} varies within a limited range under reasonable computational settings. Moreover, the relative stability of S_{total} with respect to S_{DOS} highlights the importance of incorporating the bandwidth score to ensure reliable flat-band identification. Detailed DOS curves for three representative materials under different parameter settings are shown in the bottom panel of Figure S8. As the DOS peaks near the Fermi level become more pronounced in high-scoring materials, S_{DOS} exhibits increased numerical stability, further supporting the robustness of the screening for top-ranked candidates.

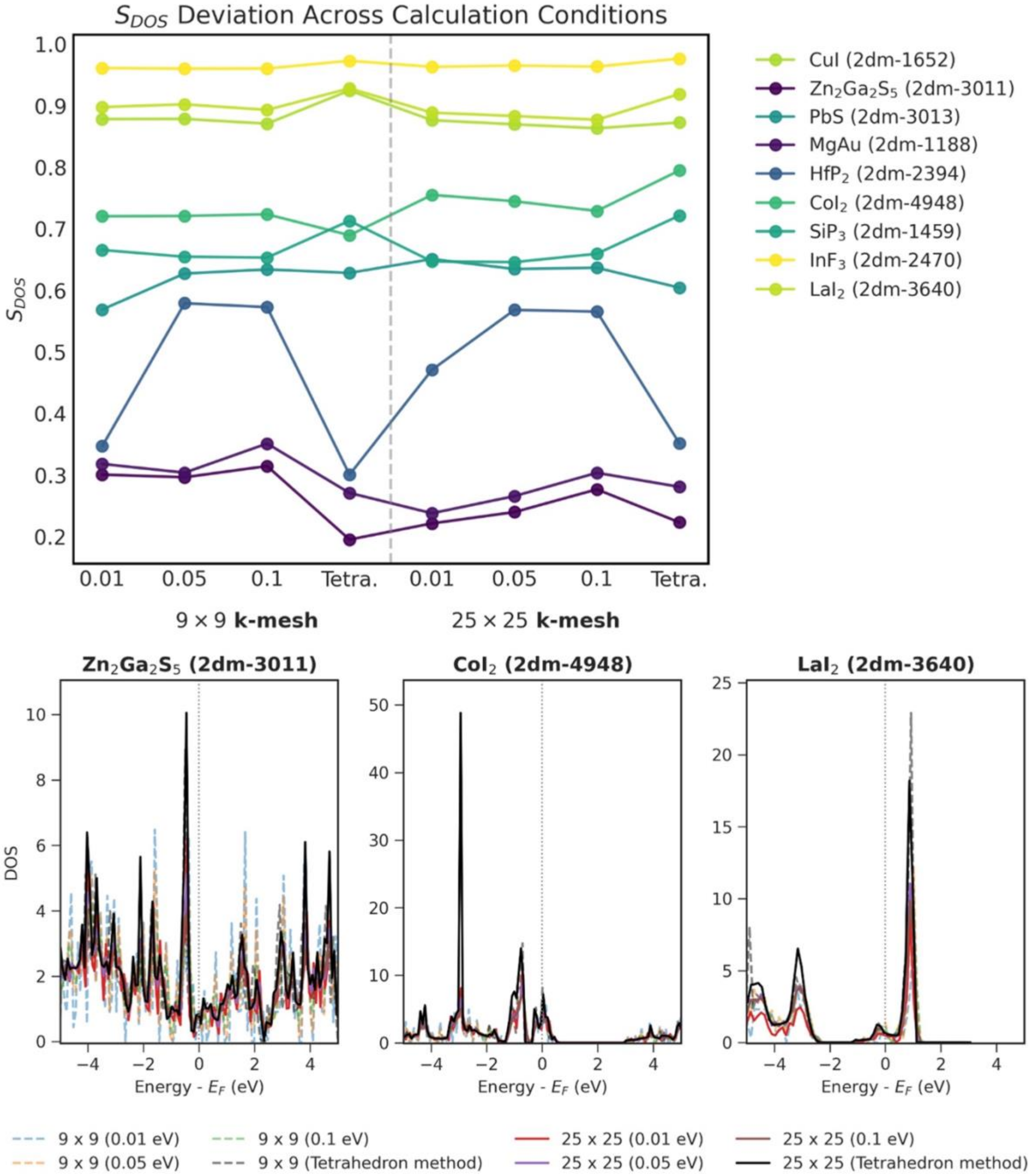

Fig. S8 Robustness analysis of the DOS score. Top: Variation of S_{DOS} under different computational conditions. Bottom: Density of states near the Fermi level for three representative materials across low-, middle-, and high-score regimes: Zn2Ga2S5 (low scores: 0.20-0.32), CoI2 (middle scores: 0.69-0.80), and LaI2 (high scores: 0.88-0.93). Line styles distinguish k-point density (dashed vs. solid), and colors indicate different smearing parameters.

REFERENCES

1. Y. Cao, V. Fatemi, S. Fang, K. Watanabe, T. Taniguchi, E. Kaxiras, P. Jarillo-Herrero, Unconventional superconductivity in magic-angle graphene superlattices. *Nature* **556**, 43–50 (2018).

2. L. Balents, C. R. Dean, D. K. Efetov, A. F. Young, Superconductivity and strong correlations in moiré flat bands. *Nat. Phys.* **16**, 725–733 (2020).

3. V. Peri, Z.-D. Song, B. A. Bernevig, S. D. Huber, Fragile topology and flat-band superconductivity in the strong-coupling regime. *Phys. Rev. Lett.* **126**, 027002 (2021).

4. Z. Lin, J.-H. Choi, Q. Zhang, W. Qin, S. Yi, P. Wang, L. Li, Y. Wang, H. Zhang, Z. Sun, L. Wei, S. Zhang, T. Guo, Q. Lu, J.-H. Cho, C. Zeng, Z. Zhang, Flatbands and emergent ferromagnetic ordering in Fe_3Sn_2 kagome lattices. *Phys. Rev. Lett.* **121**, 096401 (2018).

5. T. Neupert, L. Santos, C. Chamon, C. Mudry, Fractional quantum Hall states at zero magnetic field. *Phys. Rev. Lett.* **106**, 236804 (2011).

6. D. Sheng, Z.-C. Gu, K. Sun, L. Sheng, Fractional quantum Hall effect in the absence of Landau levels. *Nat. Commun.* **2**, 389 (2011).

7. S. A. Parameswaran, R. Roy, S. L. Sondhi, Fractional quantum Hall physics in topological flat bands. *C. R. Phys.* **14**, 816–839 (2013).

8. Z. Liu, F. Liu, Y.-S. Wu, Exotic electronic states in the world of flat bands: From theory to material. *Chin. Phys. B* **23**, 077308 (2014).

9. S. Peotta, P. Törmä, Superfluidity in topologically nontrivial flat bands. *Nat. Commun.* **6**, 8944 (2015).

10. Z. Zhang, J. Dai, C. Wang, H. Zhu, F. Pang, Z. Cheng, W. Ji, 2D kagome materials: Theoretical insights, experimental realizations, and electronic structures. *Adv. Funct. Mater.* **35**, 2416508 (2025).

11. S. Mukherjee, A. Spracklen, D. Choudhury, N. Goldman, P. Öhberg, E. Andersson, R. R. Thomson, Observation of a localized flat-band state in a photonic Lieb lattice. *Phys. Rev. Lett.* **114**, 245504 (2015).

12. A. Bhattacharya, B. Pal, Flat bands and nontrivial topological properties in an extended Lieb lattice. *Phys. Rev. B* **100**, 235145 (2019).

13. R. Bistritzer, A. H. MacDonald, Moiré bands in twisted double-layer graphene. *Proc. Natl. Acad. Sci. U.S.A.* **108**, 12233–12237 (2011).

14. M. Yankowitz, S. Chen, H. Polshyn, Y. Zhang, K. Watanabe, T. Taniguchi, D. Graf, A. F. Young, C. R. Dean, Tuning superconductivity in twisted bilayer graphene. *Science* **363**, 1059–1064 (2019).

15. G. Tarnopolsky, A. J. Kruchkov, A. Vishwanath, Origin of magic angles in twisted bilayer graphene. *Phys. Rev. Lett.* **122**, 106405 (2019).

16. F. He, Y. Zhou, Z. Ye, S. H. Cho, J. Jeong, X. Meng, Y. Wang, Moiré patterns in 2D materials: A review. *ACS Nano* **15**, 5944–5958 (2021).

17. G. Chen, A. L. Sharpe, E. J. Fox, Y. H. Zhang, S. Wang, L. Jiang, B. Lyu, H. Li, K. Watanabe, T. Taniguchi, Z. Shi, T. Senthil, D. Goldhaber-Gordon, Y. Zhang, F. Wang, Tunable correlated Chern insulator and ferromagnetism in a moiré superlattice. *Nature* **579**, 56–61 (2020).

18. S. Carr, S. Fang, E. Kaxiras, Electronic-structure methods for twisted moiré layers. *Nat. Rev. Mater.* **5**, 748–763 (2020).

19. Y. Shi, S. Xu, Y. Yang, S. Slizovskiy, S. V. Morozov, S. K. Son, S. Ozdemir, C. Mullan, J. Barrier, J. Yin, A. I. Berdyugin, B. A. Piot, T. Taniguchi, K. Watanabe, V. I. Fal’ko, K. S. Novoselov, A. K. Geim, A. Mishchenko, Electronic phase separation in multilayer rhombohedral graphite. *Nature* **584**, 210–214 (2020).

20. F. Zhang, B. Sahu, H. Min, A. H. MacDonald, Band structure of *ABC*-stacked graphene trilayers. *Phys. Rev. B* **82**, 035409 (2010).

21. Y. Park, Y. Kim, B. L. Chittari, J. Jung, Topological flat bands in rhombohedral tetralayer and multilayer graphene on hexagonal boron nitride moiré superlattices. *Phys. Rev. B* **108**, 155406 (2023).

22. A. Mishchenko, A. Bhattacharya, X. Wang, H. K. Pentz, Y. Wei, Q. Yang, Deep learning methods for 2D material electronic properties. *Digit. Discov.* **5**, 28–63 (2026).

23. H. Liu, S. Meng, F. Liu, Screening two-dimensional materials with topological flat bands. *Phys. Rev. Mater.* **5**, 084203 (2021).

24. J. Zhou, L. Shen, M. D. Costa, K. A. Persson, S. P. Ong, P. Huck, Y. Lu, X. Ma, Y. Chen, H. Tang, Y. P. Feng, 2DMatPedia, an open computational database of two-dimensional materials from top-down and bottom-up approaches. *Sci. Data* **6**, 86 (2019).

25. N. Regnault, Y. Xu, M. R. Li, D. S. Ma, M. Jovanovic, A. Yazdani, S. S. P. Parkin, C. Felser, L. M. Schoop, N. P. Ong, R. J. Cava, L. Elcoro, Z. D. Song, B. A. Bernevig, Catalogue of flat-band stoichiometric materials. *Nature* **603**, 824–828 (2022).

26. J. Duan, D. S. Ma, R. W. Zhang, W. Jiang, Z. Zhang, C. Cui, Z. M. Yu, Y. Yao, Cataloging high-quality two-dimensional van der Waals materials with flat bands. *Adv. Funct. Mater.* **34**, 2313067 (2024).

27. M. G. Vergniory, L. Elcoro, C. Felser, N. Regnault, B. A. Bernevig, Z. Wang, A complete catalogue of high-quality topological materials. *Nature* **566**, 480–485 (2019).

28. M. G. Vergniory, B. J. Wieder, L. Elcoro, S. S. P. Parkin, C. Felser, B. A. Bernevig, N. Regnault, All topological bands of all nonmagnetic stoichiometric materials. *Science* **376**, eabg9094 (2022).

29. A. Bhattacharya, I. Timokhin, R. Chatterjee, Q. Yang, A. Mishchenko, Deep learning approach to genome of two-dimensional materials with flat electronic bands. *npj Comput. Mater.* **9**, 101 (2023).

30. P. M. Neves, J. P. Wakefield, S. Fang, H. Nguyen, L. Ye, J. G. Checkelsky, Crystal net catalog of model flat band materials. *npj Comput. Mater.* **10**, 39 (2024).

31. Y. Zhang, W. Xu, G. Liu, Z. Zhang, J. Zhu, M. Li, Bandgap prediction of two-dimensional materials using machine learning. *PLOS ONE* **16**, e0255637 (2021).

32. A. C. Rajan, A. Mishra, S. Satsangi, R. Vaish, H. Mizuseki, K. R. Lee, A. K. Singh, Machine-learning-assisted accurate band gap predictions of functionalized MXene. *Chem. Mater.* **30**, 4031–4038 (2018).

33. N. R. Knøsgaard, K. S. Thygesen, Representing individual electronic states for machine learning GW band structures of 2D materials. *Nat. Commun.* **13**, 468 (2022).

34. Y. Dong, C. Wu, C. Zhang, Y. Liu, J. Cheng, J. Lin, Bandgap prediction by deep learning in configurationally hybridized graphene and boron nitride. *npj Comput. Mater.* **5**, 26 (2019).

35. W. Gong, T. Sun, H. Bai, J.-Y. Tsai, H. Ling, Q. Yan, Graph transformer networks for accurate band structure prediction: An end-to-end approach. arXiv:2411.16483 [cond-mat.mtrl-sci] (2024).

36. Y.-T. Yeh, J. Ock, A. B. Farimani, Text to band gap: Pre-trained language models as encoders for semiconductor band gap prediction. arXiv:2501.03456 [cs.CL] (2025).

37. J. Lee, C. Park, H. Yang, S. Lim, S. Han, CAST: Cross attention based multimodal fusion of structure and text for materials property prediction. arXiv:2502.06836 [cs.LG] (2025).

38. T. T. Heikkilä, N. B. Kopnin, G. E. Volovik, Flat bands in topological media. *JETP Lett.* **94**, 233–239 (2011).

39. Z. Li, J. Zhuang, L. Wang, H. Feng, Q. Gao, X. Xu, W. Hao, X. Wang, C. Zhang, K. Wu, S. X. Dou, L. Chen, Z. Hu, Y. Du, Realization of flat band with possible nontrivial topology in electronic Kagome lattice. *Sci. Adv.* **4**, eaau4511 (2018).

40. K. Choudhary, B. DeCost, Atomistic line graph neural network for improved materials property predictions. *npj Comput. Mater.* **7**, 185 (2021).

41. Y. Liu, M. Ott, N. Goyal, J. Du, M. Joshi, D. Chen, O. Levy, M. Lewis, L. Zettlemoyer, V. Stoyanov, RoBERTa: A robustly optimized bert pretraining approach. arXiv:1907.11692 [cs.CL] (2019).

42. L. McInnes, J. Healy, J. Melville, UMAP: Uniform manifold approximation and projection for dimension reduction. arXiv:1802.03426 [stat.ML] (2020).

43. M. N. Gjerding, A. Taghizadeh, A. Rasmussen, S. Ali, F. Bertoldo, T. Deilmann, N. R. Knøsgaard, M. Kruse, A. H. Larsen, S. Manti, T. G. Pedersen, U. Petralanda, T. Skovhus, M. K. Svendsen, J. J. Mortensen, T. Olsen, K. S. Thygesen, Recent progress of the computational 2D materials database (C2DB). *2D Mater.* **8**, 044002 (2021).

44. U. Petralanda, Y. Jiang, B. A. Bernevig, N. Regnault, L. Elcoro, Two-dimensional topological quantum chemistry and catalog of topological materials. arXiv:2411.08950 [cond-mat.mes-hall] (2024).

45. B. Bradlyn, L. Elcoro, J. Cano, M. G. Vergniory, Z. Wang, C. Felser, M. I. Aroyo, B. A. Bernevig, Topological quantum chemistry. *Nature* **547**, 298–305 (2017).

46. Z. Song, Z. Wang, W. Shi, G. Li, C. Fang, B. A. Bernevig, All magic angles in twisted bilayer graphene are topological. *Phys. Rev. Lett.* **123**, 036401 (2019).

47. Z. Sun, H. Zhou, C. Wang, S. Kumar, D. Geng, S. Yue, X. Han, Y. Haraguchi, K. Shimada, P. Cheng, L. Chen, Y. Shi, K. Wu, S. Meng, B. Feng, Observation of topological flat bands in the kagome semiconductor Nb3Cl8. *Nano Lett.* **22**, 4596–4602 (2022).

48. F. Nogueira, Bayesian optimization: Open source constrained global optimization tool for Python (2014); https://github.com/fmfn/BayesianOptimization.

49. L. McInnes, J. Healy, S. Astels, hdbscan: Hierarchical density based clustering. *J. Open Source Softw.* **2**, 205 (2017).

50. A. Paszke, Pytorch: An imperative style, high-performance deep learning library. arXiv:1912.01703 [cs.LG] (2019).

51. M. Y. Wang, "Deep graph library: Towards efficient and scalable deep learning on graphs," in *ICLR Workshop on Representation Learning on Graphs and Manifolds* (2019).

52. F. Pedregosa, G. Varoquaux, A. Gramfort, V. Michel, B. Thirion, O. Grisel, M. Blondel, P. Prettenhofer, R. Weiss, V. Dubourg, Scikit-learn: Machine learning in Python. *J. Mach. Learn. Res.* **12**, 2825–2830 (2011).

53. S. P. Ong, W. D. Richards, A. Jain, G. Hautier, M. Kocher, S. Cholia, D. Gunter, V. L. Chevrier, K. A. Persson, G. Ceder, Python Materials Genomics (pymatgen): A robust, open-source python library for materials analysis. *Comput. Mater. Sci.* **68**, 314–319 (2013).

54. P. Virtanen, R. Gommers, T. E. Oliphant, M. Haberland, T. Reddy, D. Cournapeau, E. Burovski, P. Peterson, W. Weckesser, J. Bright, S. J. van der Walt, M. Brett, J. Wilson, K. J. Millman, N. Mayorov, A. R. J. Nelson, E. Jones, R. Kern, E. Larson, C. J. Carey, İ. Polat, Y. Feng, E. W. Moore, J. VanderPlas, SciPy 1.0 Contributors, SciPy 1.0: Fundamental algorithms for scientific computing in Python. *Nat. Methods* **17**, 261–272 (2020).

55. T. Wolf, L. Debut, V. Sanh, J. Chaumond, C. Delangue, A. Moi, P. Cistac, T. Rault, R. Louf, M. Funtowicz, J. Davison, S. Shleifer, P. von Platen, C. Ma, Y. Jernite, J. Plu, C. Xu, T. L. Scao, S. Gugger, M. Drame, Q. Lhoest, A. M. Rush, "Transformers: State-of-the-art natural language processing," in *Proceedings of the 2020 Conference on Empirical Methods in Natural Language Processing: System Demonstrations,* Association for Computational Linguistics (ACL) (2020), pp. 38–45.

56. J. P. Perdew, K. Burke, M. Ernzerhof, Generalized gradient approximation made simple. *Phys. Rev. Lett.* **77**, 3865–3868 (1996).

57. A. Jain, G. Hautier, C. J. Moore, S. Ping Ong, C. C. Fischer, T. Mueller, K. A. Persson, G. Ceder, A high-throughput infrastructure for density functional theory calculations. *Comput. Mater. Sci.* **50**, 2295–2310 (2011).

58. J. Gao, Q. Wu, C. Persson, Z. Wang, Irvsp: To obtain irreducible representations of electronic states in the VASP. *Comput. Phys. Commun.* **261**, 107760 (2021).

59. A. Togo, L. Chaput, T. Tadano, I. Tanaka, Implementation strategies in phonopy and phono3py. *J. Phys. Condens. Matter* **35**, 353001 (2023).

60. A. Togo, First-principles phonon calculations with phonopy and phono3py. *J. Phys. Soc. Jpn.* **92**, 012001 (2023).

61. J. Gao, Y. Qian, H. Jia, Z. Guo, Z. Fang, M. Liu, H. Weng, Z. Wang, Unconventional materials: The mismatch between electronic charge centers and atomic positions. *Sci. Bull.* **67**, 598–608 (2022).

62. S. Nie, Y. Qian, J. Gao, Z. Fang, H. Weng, Z. Wang, Application of topological quantum chemistry in electrides. *Phys. Rev. B* **103**, 205133 (2021).

63. M. I. Aroyo, J. M. Perez-Mato, D. Orobengoa, E. Tasci, G. de la Flor, A. Kirov, Crystallography online: Bilbao crystallographic server. *Bulg. Chem. Commun* **43**, 183–197 (2011).

64. M. I. Aroyo, J. M. Perez-Mato, C. Capillas, E. Kroumova, S. Ivantchev, G. Madariaga, A. Kirov, H. Wondratschek, Bilbao Crystallographic Server: I. Databases and crystallographic computing programs. *Z. Kristallogr. Cryst. Mater.* **221**, 15–27 (2006).

65. M. I. Aroyo, A. Kirov, C. Capillas, J. M. Perez-Mato, H. Wondratschek, Bilbao Crystallographic Server. II. Representations of crystallographic point groups and space groups. *Acta Crystallogr. A* **62**, 115–128 (2006).

66. U. Herath, P. Tavadze, X. He, E. Bousquet, S. Singh, F. Muñoz, A. H. Romero, PyProcar: A Python library for electronic structure pre/post-processing. *Comput. Phys. Commun.* **251**, 107080 (2020).

67. L. Lang, P. Tavadze, A. Tellez, E. Bousquet, H. Xu, F. Muñoz, N. Vasquez, U. Herath, A. H. Romero, Expanding PyProcar for new features, maintainability, and reliability. *Comput. Phys. Commun.* **297**, 109063 (2024).

68. S. Durdy, M. W. Gaultois, V. V. Gusev, D. Bollegala, M. J. Rosseinsky, Random projections and kernelised leave one cluster out cross validation: Universal baselines and evaluation tools for supervised machine learning of material properties. *Digit. Discov.* **1**, 763–778 (2022).
